# Synthesis and crystal structure of peptide dimethyl biphenyl hybrid C_52_H_60_N_6_O_10_·0.25H_2_O

**DOI:** 10.1107/S2056989020012931

**Published:** 2020-09-25

**Authors:** Xuan Tu Nguyen, Thuy Quynh Le, Tra My Bui Thi, Dinh Hung Mac, Thai Thanh Thu Bui

**Affiliations:** a Department of Chemistry, VNU University of science, Vietnam National University, Hanoi, 19 Le Thanh Tong, Hanoi, Vietnam

**Keywords:** crystal structure, hydrogen bonding, peptide dimethyl biphenyl hybrids, Pro-Phe-Ala

## Abstract

The synthesis and crystal structure of peptide 6,6′-dimethyl biphenyl hybrid are described. The title compound was synthesized by reaction between 6,6′-dimethyl-[1,1′-biphen­yl]-2,2′-dicarbonyl dichloride in CH_2_Cl_2_, amine HN–proline–phenyl­alanine–alanine–COOMe and Et_3_N at 273 K under N_2_ atmosphere and characterized by single-crystal X-ray diffraction. The asymmetric unit contains one peptide mol­ecule and a quarter of a water mol­ecule. A disorder of a methyl and meth­oxy­carbonyl group of one alanine residue is observed with occupancy ratio 0.502 (6):0.498 (6). The structure is consolidated by intra- and inter­molecular hydrogen bonds.

## Abstract 2

The crystal structure of the title compound shows a disorder of the methyl and meth­oxy­carbonyl groups of one alanine residue. Compared to previously reported peptide biphenyl hybrids, the backbone torsion angles are different.

## Chemical context   

Since the first application in 1922 of peptides in the treatment of diabetes with insulin (Banting *et al.*, 1922 ▸), the chemistry of peptides has become a very important domain in the search of new therapeutic drugs. From 2011 to 2018, the global market of drugs has increased from US $ 14.1 to 24.4 billion. With more than 140 peptides in clinical trials, the number of peptide-based drugs is expected to grow significantly (Fosgerau *et al.*, 2015 ▸). Despite their tremendous potential, applications of peptides for pharmaceutical purposes are limited by their instability toward enzymatic systems, short half-life, rapid renal clearance, and formulation challenges (Otvos *et al.*, 2014 ▸). These problems can be overcome by modifying the linear peptide to enhance the stability and therefore the selectivity and affinity. The biphenyl structure is present in numerous pharmaceuticals and bioactive compounds, as illustrated by the glycopeptide anti­biotic vancomycin, the proteasome inhibitor TMC-95A (Kaiser *et al.*, 2004 ▸) and aryl­omycins (Schimana *et al.*, 2002 ▸). A statistical analysis of NMR data indicates that compounds containing the biphenyl structure can bind a wide range of proteins with high levels of specificity (Hajduk *et al.*, 2000 ▸). Coupling of a small protein chain to the biphenyl structure is a strategy to create a new family of peptidomimetic compounds, which can be used in medicinal chemistry because of its specific conformation and its particular hydrogen-bonding inter­actions.

**Figure 1:**
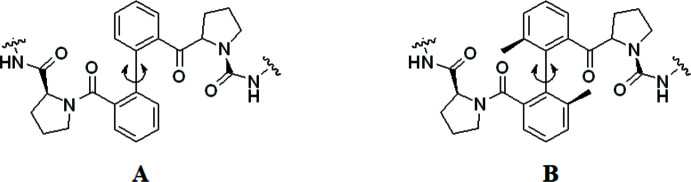
Peptide–biphenyl hybrids A and B.

The synthesis and biological activity as calpain inhibitor of peptide–biphenyl hybrids type I have been reported by Montero and Mann (Montero *et al.*, 2004*a* ▸,*b* ▸; Mann *et al.*, 2002 ▸). Amine *et al.* (2002 ▸) synthesized a bis amido–copper(II) complex from N-containing tetra­dentate ligands having two amido groups with a biphenyl skeleton, which is used as a DNA cleaving agent. Recently, we have reported crystallographic studies of a peptide-biphenyl hybrid A (Fig. 1 ▸) with tripeptide Pro–Phe–Ala (Le *et al.*, 2020 ▸).
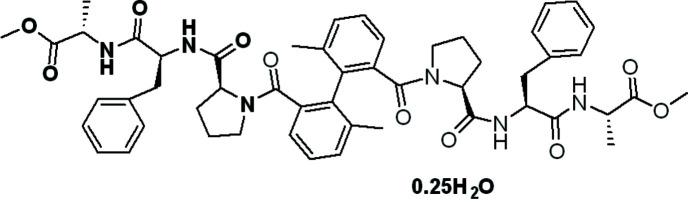


We report herein the synthesis and crystallographic study of a peptide-2,2′-biphenyl B (Fig. 1 ▸) with the introduction of two methyl groups at the 6-6′ positions to prevent free rotation around the central ar­yl–aryl bond.

## Structural commentary   

**Figure 2:**
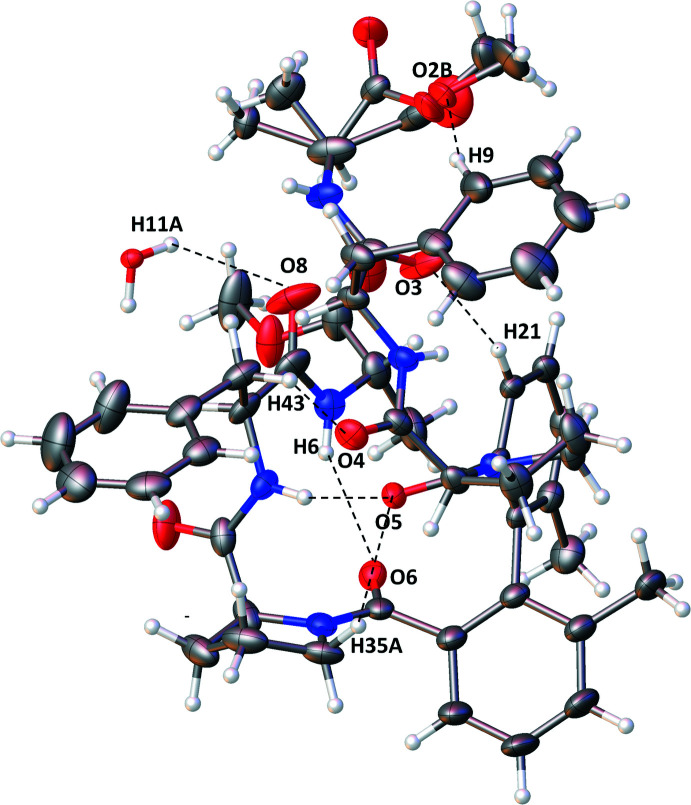
A view of the mol­ecular structure of the title compound showing displacement ellipsoids drawn at the 50% probability level and hydrogen bonds (dashed lines) within the asymmetric unit. H atoms are shown as small circles of arbitrary radii.

The compound dimethyl 2,2′-[((2*S*,2′*S*)-2,2′-{[(2*S*,2′*S*)-1,1′-(6,6′-dimethyl-[1,1′-biphen­yl]-2,2′-dicarbon­yl)bis­(pyrrolidine-1,2-diyl-2-carbon­yl)]bis­(aza­nedi­yl)}bis­(3-phenyl­propano­yl))bis­(aza­nedi­yl)](2*S*,2′*S*)-dipropionate (Fig. 2 ▸) crystallizes in the monoclinic space group *C*2 with one mol­ecule of peptide biphenyl hybrid accompanied by a quarter of a water mol­ecule in the asymmetric unit. Two methyl groups have been introduced to the biphenyl rings at the 6,6′ position in order to limit the rotation of the two central phenyl rings in solution. In the solid state, the dihedral angle between biphenyl rings C20–C25 and C27-C32 is 73.8 (3)°. However, this value is similar to that of a previous compound not bearing the methyl groups (C_50_H_56_N_6_O_10_·0.5H_2_O; Le *et al.*, 2020 ▸). A disorder of a methyl and meth­oxy­carbonyl group of alanine is observed in the crystal structure and was refined with an occupancy ratio of 0.502 (6):0.498 (6).

The backbone conformation of the two tripeptide fragments is characterized by the torsion angles ω, φ, ψ (see Table 1 ▸). The torsion angles φ and ψ of amino acids Ala1, Ala2, Phe2 correspond with the usual α-helix (right-handed) region of the Ramachandran plot, and only the torsion angles of amino acid Phe1 fall into the corresponding type β-sheet Ramachandran plot region. For both prolines, the related torsion angles lie in the α region of the Ramachandran plot for proline.

There are six intra­molecular hydrogen bonds formed in the structure of the title compound (Table 2 ▸). Two hydrogen bonds are formed between the NH and CO groups with H⋯O distances of 2.07 Å for N5—H5 ⋯O5 and 2.42 Å for N6—H6⋯O6. The latter value is noticeably longer than the values observed (from 2.04 to 2.29 Å) in other reported peptides (Ranganathan *et al.*, 1997 ▸; Le *et al.*, 2020 ▸). Four other intra­molecular bonds are formed between CH and CO groups with distances from 2.35 to 2.59 Å.

## Supra­molecular features   

**Figure 3:**
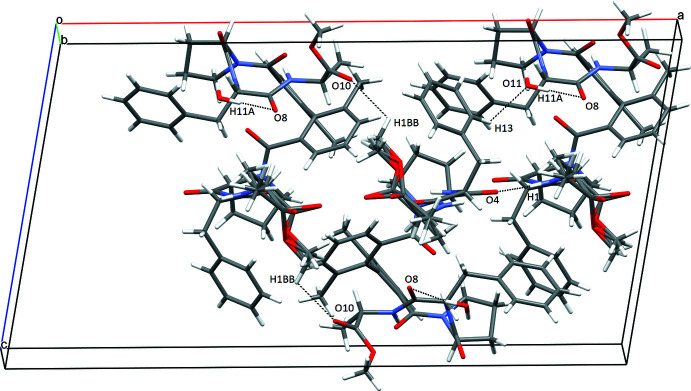
Crystal packing of the title compound, indicating some inter­molecular hydrogen bonds (dashed lines).

In the crystal, the packing is characterized by N—H⋯O, O—H⋯O and C—H⋯O hydrogen bonding (see Table 2, Fig. 3 ▸ ▸). The strongest inter­molecular inter­action is formed between NH and CO groups of two neighboring peptide residues [N1—H1⋯O4^i^, with *d* = 2.01 Å; symmetry code: (i) 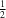 − *x*, 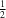 + *y*, 1 − *z*]. Furthermore, there are six additional hydrogen bonds linking the mol­ecules. Two contacts are established between the water mol­ecule and two tripeptides (O11—H11*A*⋯O8; C13— H13⋯O11). Four C—H⋯O=C contacts with H⋯O distances ranging from 2.39 to 2.60 Å further consolidate the crystal packing. In addition, the mol­ecules are linked by two inter­molecular C—H ⋯π inter­actions, one between a proline H atom and the phenyl ring of a phenyl­alanine residue, the other between a H atom of the disordered methyl group and a phenyl ring of the central biphenyl fragment.

## Database survey   

A search of the Cambridge Structural Database (version 5.41 with update of March 2020; Groom *et al.*, 2016 ▸) for peptide–dimethyl biphenyl hybrids was conducted. There are seven dimethyl biphenyl hybrid structures with only one amino acid, including JITYET (Linden & Rippert, 2018*a* ▸), JITZEU (Linden & Rippert, 2018*b* ▸), JITYOD (Linden & Rippert, 2018*c* ▸), NOSPUG & NOSQAN (Weigand & Feigel, 1998 ▸), PITSUJ (Linden *et al.*, 2018*d* ▸) and NIKJOI (Samadi *et al.*, 2013 ▸). For these structures the dihedral angles between the dimethyl biphenyl rings varies from 82.0 to 95.8^o^, larger than the corresponding angle of the title compound.

## Synthesis and crystallization   

To a round-bottom flask was added 6,6′-dimethyl-[1,1′-biphen­yl]-2,2′-di­carb­oxy­lic acid (1 eq.) and SOCl_2_ (3 eq.) respectively under a nitro­gen atmosphere. The mixture was heated under reflux for 4 h and was then evaporated under vacuum. The acid chloride was used in the next step without further purification.

To a round-bottom flask was added amine HN–proline–phenyl­alanine–alanine–COOMe (1 eq.), Et_3_N (2 eq.) and anhydrous CH_2_Cl_2_ (50mL). To this solution was added a solution of (6,6′-dimethyl-[1,1′-biphen­yl]-2,2′-dicarbonyl dichloride in CH_2_Cl_2_ at 273 K under an N_2_ atmosphere. After completion of the reaction, the mixture was washed with 1 *N* HCl solution, water and a solution of brine, respectively. The organic phase was dried over Na_2_SO_4_, filtered and evaporated under reduced pressure. The crude product was then purified by flash chromatography (AcOEt/hexane 3:2) to give a white solid (60% yield). The compound was recrystallized by slow evaporation in methanol to give crystals suitable for X-ray diffraction.

## Refinement   

Crystal data, data collection and structure refinement details are summarized in Table 3 ▸. The methyl and meth­oxy­carbonyl groups of alanine show two conformations with refined occupancy factors converging to 0.502 (6) and 0.498 (6). Geometrical restraints were applied to the disordered atoms. H atoms were placed at calculated positions (C—H = 0.95–1.08 Å and N—H = 0.88 Å), with isotropic displacement parameters *U*_iso_(H) = 1.5*U*_eq_(C) for methyl H atoms and 1.2*U*_eq_(C,N) for all other H atoms. The solvent water mol­ecule is disordered and was refined with a site occupation factor fixed to 0.25. The H atoms of the water mol­ecule were located in difference-Fourier maps and refined in riding-model approximation with *U*_iso_(H) = 1.5*U*_eq_(O).

## Supplementary Material

Crystal structure: contains datablock(s) I. DOI: 10.1107/S2056989020012931/vm2240sup1.cif

Supporting information file. DOI: 10.1107/S2056989020012931/vm2240Isup3.cdx

Supporting information file. DOI: 10.1107/S2056989020012931/vm2240Isup4.cdx

Supporting information file. DOI: 10.1107/S2056989020012931/vm2240sup5.docx

Supporting information file. DOI: 10.1107/S2056989020012931/vm2240sup6.docx

Supporting information file. DOI: 10.1107/S2056989020012931/vm2240sup7.tif

Supporting information file. DOI: 10.1107/S2056989020012931/vm2240sup8.tif

CCDC reference: 2026794

Additional supporting information:  crystallographic information; 3D view; checkCIF report

## Figures and Tables

**Table 1:** Backbone torsion angles ω, φ, ψ (°) for the two tripeptide fragments

C20—C19—N3—C15	178.3 (2)	C32—C34—N4—C38	−164.6 (2)
C19—N3—C15—C14	−73.4 (3)	C34—N4—C38—C39	−69.1 (3)
N3—C15—C14—N2	−17.5 (3)	N4—C38—C39—N5	−14.4 (4)
C15—C14—N2—C6	176.5 (2)	C38—C39—N5—C40	−177.2 (2)
C14—N2—C6—C5	−163.0 (2)	C39—N5—C40—C48	−106.8 (3)
N2—C6—C5—N1	171.4 (2)	N5—C40—C48—N6	18.6 (3)
C6—C5—N1—C3	−174.8 (3)	C40—C48—N6—C49	179.1 (2)
C5—N1—C3—C2*B*	−58.0 (5)	C48—N6—C49—C51	−60.9 (3)
N1—C3—C2*B*—O2*B*	−39.6 (13)	N6—C49—C51—O9	−35.0 (4)

**Table 2:** Hydrogen-bond geometry (Å, °) *Cg*3 and *Cg*5 are the centroids of the C8–C13 and C27–C32 rings, respectively.

*D*—H⋯*A*	*D*—H	H⋯*A*	*D*⋯*A*	*D*—H⋯*A*
N5—H5⋯O5	0.88	2.07	2.923 (3)	162
N6—H6⋯O6	0.88	2.42	3.233 (3)	154
C9—H9⋯O2*B*	0.95	2.35	3.270 (18)	164
C21—H21⋯O3	0.95	2.44	3.352 (4)	161
C35—H35*A*⋯O5	0.99	2.51	3.171 (4)	124
C43—H43⋯O4	0.95	2.59	3.443 (4)	149
N1—H1⋯O4^i^	0.88	2.01	2.865 (3)	163
C1*B*—H1*BB*⋯O10^ii^	0.98	2.46	2.913 (16)	108
C30—H30⋯O8^iii^	0.95	2.46	3.222 (4)	137
C35—H35⋯O7^iv^	0.99	2.39	3.228 (4)	142
C52—H52*B*⋯O10^v^	0.98	2.60	3.559 (5)	166
O11—H11*A*⋯O8	0.87	2.48	3.136 (6)	133
C13—H13⋯O11^vi^	0.95	2.52	3.155 (7)	124
C36—H36*B*⋯*Cg*3^vi^	0.99	2.94	3.845 (4)	152
C4*A*—H4*AC*⋯*Cg*5^vii^	1.05 (8)	2.93 (7)	3.770 (8)	135 (5)

Symmetry codes: (i) 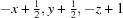; (ii) 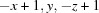; (iii) 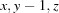; (iv) 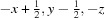; (v) 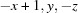; (vi) 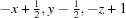; (vii) 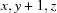.

**Table 3:** Experimental details

Crystal data	
Chemical formula	C_52_H_60_N_6_O_10_·0.25H_2_O
*M*_r_	933.56
Crystal system, space group	Monoclinic, *C*2
Temperature (K)	100
*a*, *b*, *c* (Å)	27.505 (3), 12.3814 (12), 14.6346 (14)
β (°)	99.999 (3)
*V* (Å^3^)	4908.2 (8)
*Z*	4
Radiation type	Mo *K*α
μ (mm^−1^)	0.09
Crystal size (mm)	0.3 × 0.2 × 0.1

Data collection	
Diffractometer	Bruker D8 Quest CMOS
Absorption correction	Multi-scan (*SADABS*-; Bruker, 2013 ▸)
*T*_min_, *T*_max_	0.713, 0.745
No. of measured, independent and observed [*I* > 2σ(*I*)] reflections	84318, 9371, 7959
*R*_int_	0.062
(sin θ/λ)_max_ (Å^−1^)	0.611

Refinement	
*R*[*F* ^2^ > 2σ(*F* ^2^)], *wR*(*F* ^2^), *S*	0.038, 0.086, 1.06
No. of reflections	9371
No. of parameters	693
No. of restraints	4
H-atom treatment	H atoms treated by a mixture of independent and constrained refinement
Δρ_max_, Δρ_min_ (e Å^−3^)	0.28, −0.16
Absolute structure	Flack *x* determined using 3323 quotients [(*I* ^+^)−(*I* ^−^)]/[(*I* ^+^)+(*I* ^−^)] (Parsons et al., 2013 ▸)
Absolute structure parameter	−0.1 (3)

Computer programs: *APEX22* and *SAINT* (Bruker, 2013 ▸), *SHELXT* (Sheldrick, 2015*a* ▸), *SHELXL* (Sheldrick, 2015*b* ▸) and *OLEX2* (Dolomanov *et al.*, 2009 ▸).

## supplementary crystallographic information

### Crystal data

**Table:**

C_52_H_60_N_6_O_10_·0.25H_2_O	*F*(000) = 1986
*M**_r_* = 933.56	*D*_x_ = 1.263 Mg m^−^^3^
Monoclinic, *C*2	Mo *K*α radiation, λ = 0.71073 Å
*a* = 27.505 (3) Å	Cell parameters from 9371 reflections
*b* = 12.3814 (12) Å	θ = 2.8–25.8°
*c* = 14.6346 (14) Å	µ = 0.09 mm^−^^1^
β = 99.999 (3)°	*T* = 100 K
*V* = 4908.2 (8) Å^3^	Needle, clear light colourless
*Z* = 4	0.3 × 0.2 × 0.1 mm

### Data collection

**Table:**

Bruker D8 Quest CMOS diffractometer	7959 reflections with *I* > 2σ(*I*)
φ and ω scans	*R*_int_ = 0.062
Absorption correction: multi-scan (SADABS-; Bruker, 2013)	θ_max_ = 25.8°, θ_min_ = 2.8°
*T*_min_ = 0.713, *T*_max_ = 0.745	*h* = −33→33
84318 measured reflections	*k* = −15→15
9371 independent reflections	*l* = −17→17

### Refinement

**Table:**

Refinement on *F*^2^	Hydrogen site location: mixed
Least-squares matrix: full	H atoms treated by a mixture of independent and constrained refinement
*R*[*F*^2^ > 2σ(*F*^2^)] = 0.038	*w* = 1/[σ^2^(*F*_o_^2^) + (0.0359*P*)^2^ + 2.1291*P*] where *P* = (*F*_o_^2^ + 2*F*_c_^2^)/3
*wR*(*F*^2^) = 0.086	(Δ/σ)_max_ < 0.001
*S* = 1.06	Δρ_max_ = 0.28 e Å^−^^3^
9371 reflections	Δρ_min_ = −0.16 e Å^−^^3^
693 parameters	Absolute structure: Flack *x* determined using 3323 quotients [(*I*^+^)-(*I*^-^)]/[(*I*^+^)+(*I*^-^)] (Parsons et al., 2013)
4 restraints	Absolute structure parameter: −0.1 (3)
Primary atom site location: dual	

### Special details

**Table:**

Geometry. All esds (except the esd in the dihedral angle between two l.s. planes) are estimated using the full covariance matrix. The cell esds are taken into account individually in the estimation of esds in distances, angles and torsion angles; correlations between esds in cell parameters are only used when they are defined by crystal symmetry. An approximate (isotropic) treatment of cell esds is used for estimating esds involving l.s. planes.

### Fractional atomic coordinates and isotropic or equivalent isotropic displacement parameters (Å^2^)

**Table:**

	*x*	*y*	*z*	*U*_iso_*/*U*_eq_	Occ. (<1)
O5	0.33318 (6)	0.37671 (15)	0.30911 (12)	0.0262 (4)	
O4	0.25033 (7)	0.41245 (17)	0.49754 (15)	0.0367 (5)	
O6	0.37283 (7)	0.35432 (16)	0.07943 (13)	0.0314 (4)	
N3	0.36969 (8)	0.32535 (18)	0.45153 (14)	0.0252 (5)	
N2	0.32176 (8)	0.50351 (19)	0.50273 (15)	0.0291 (5)	
H2	0.3537	0.4960	0.5038	0.035*	
O7	0.25946 (8)	0.5451 (2)	−0.00116 (15)	0.0531 (6)	
O3	0.38550 (8)	0.64671 (17)	0.4803 (2)	0.0552 (7)	
O9	0.40799 (7)	0.7684 (2)	0.02936 (16)	0.0536 (7)	
N1	0.33544 (8)	0.7906 (2)	0.48045 (17)	0.0351 (6)	
H1	0.3070	0.8150	0.4917	0.042*	
N5	0.29721 (8)	0.50901 (18)	0.14594 (15)	0.0277 (5)	
H5	0.3085	0.4583	0.1862	0.033*	
N4	0.29975 (8)	0.2897 (2)	0.10755 (15)	0.0302 (5)	
O2A	0.4152 (8)	0.8404 (16)	0.5865 (9)	0.052 (4)	0.498 (6)
O10	0.47865 (9)	0.8022 (2)	0.12726 (19)	0.0665 (8)	
C19	0.37162 (9)	0.35712 (19)	0.36445 (17)	0.0221 (5)	
O8	0.36763 (10)	0.74704 (19)	0.2248 (2)	0.0667 (8)	
N6	0.38529 (8)	0.6049 (2)	0.14123 (16)	0.0335 (5)	
H6	0.3742	0.5463	0.1106	0.040*	
C20	0.42128 (9)	0.3761 (2)	0.33809 (17)	0.0224 (5)	
C14	0.29481 (10)	0.4139 (2)	0.49355 (18)	0.0277 (6)	
C22	0.49273 (10)	0.4901 (2)	0.34994 (19)	0.0300 (6)	
H22	0.5116	0.5499	0.3769	0.036*	
C21	0.44813 (10)	0.4663 (2)	0.37539 (19)	0.0264 (6)	
H21	0.4355	0.5113	0.4184	0.032*	
C39	0.27589 (10)	0.4803 (3)	0.05996 (19)	0.0336 (7)	
C34	0.34964 (10)	0.2867 (2)	0.11713 (18)	0.0292 (6)	
C25	0.43896 (10)	0.3099 (2)	0.27349 (17)	0.0264 (6)	
C15	0.32173 (10)	0.3090 (2)	0.48016 (18)	0.0290 (6)	
H15	0.3004	0.2638	0.4327	0.035*	
C23	0.50992 (10)	0.4261 (2)	0.2846 (2)	0.0339 (7)	
H23	0.5403	0.4441	0.2659	0.041*	
C32	0.37479 (11)	0.1926 (2)	0.1698 (2)	0.0342 (7)	
C5	0.34545 (10)	0.6858 (2)	0.4894 (2)	0.0362 (7)	
C42	0.22491 (12)	0.6148 (3)	0.2431 (2)	0.0393 (7)	
C8	0.32288 (11)	0.5941 (3)	0.6886 (2)	0.0368 (7)	
C18	0.41107 (10)	0.2861 (3)	0.52286 (19)	0.0355 (7)	
H18A	0.4391	0.3375	0.5316	0.043*	
H18B	0.4228	0.2143	0.5062	0.043*	
C24	0.48386 (10)	0.3365 (2)	0.24582 (19)	0.0326 (7)	
C6	0.30370 (9)	0.6123 (2)	0.5111 (2)	0.0303 (6)	
H6A	0.2743	0.6231	0.4612	0.036*	
C28	0.43711 (12)	0.1101 (2)	0.2858 (2)	0.0382 (7)	
C38	0.27062 (10)	0.3606 (3)	0.03950 (19)	0.0348 (7)	
H38	0.2789	0.3459	−0.0232	0.042*	
C27	0.41520 (10)	0.2032 (2)	0.2421 (2)	0.0306 (6)	
C7	0.28776 (10)	0.6356 (3)	0.6048 (2)	0.0360 (7)	
H7A	0.2831 (11)	0.715 (3)	0.610 (2)	0.043*	
H7B	0.2561 (12)	0.604 (3)	0.606 (2)	0.043*	
C40	0.30215 (11)	0.6212 (2)	0.1744 (2)	0.0344 (7)	
H40	0.2820	0.6644	0.1237	0.041*	
C48	0.35480 (12)	0.6626 (2)	0.1841 (2)	0.0395 (7)	
C51	0.44291 (12)	0.7469 (3)	0.1020 (2)	0.0465 (8)	
C41	0.27949 (12)	0.6391 (2)	0.2612 (2)	0.0403 (7)	
H41A	0.2962	0.5918	0.3116	0.048*	
H41B	0.2848	0.7150	0.2818	0.048*	
C49	0.43632 (11)	0.6369 (3)	0.1441 (2)	0.0381 (7)	
H49	0.4534	0.6375	0.2103	0.046*	
C31	0.35836 (13)	0.0900 (2)	0.1390 (2)	0.0456 (8)	
H31	0.3315	0.0830	0.0888	0.055*	
C17	0.38721 (11)	0.2796 (3)	0.6093 (2)	0.0461 (8)	
H17A	0.3879	0.3506	0.6406	0.055*	
H17B	0.4040	0.2256	0.6538	0.055*	
C43	0.20546 (13)	0.5304 (3)	0.2867 (2)	0.0406 (8)	
H43	0.2267	0.4876	0.3304	0.049*	
C26	0.50273 (12)	0.2713 (3)	0.1714 (2)	0.0459 (8)	
H26A	0.5078	0.1962	0.1920	0.069*	
H26B	0.5341	0.3018	0.1604	0.069*	
H26C	0.4785	0.2737	0.1139	0.069*	
C35	0.26820 (12)	0.2261 (3)	0.1605 (2)	0.0403 (8)	
H35A	0.2818	0.2267	0.2278	0.048*	
H35B	0.2648	0.1504	0.1386	0.048*	
C9	0.36689 (11)	0.6476 (3)	0.7211 (3)	0.0515 (9)	
H9	0.3753	0.7096	0.6890	0.062*	
C30	0.38083 (14)	−0.0007 (3)	0.1809 (3)	0.0563 (10)	
H30	0.3697	−0.0703	0.1592	0.068*	
C36	0.21906 (12)	0.2852 (3)	0.1399 (2)	0.0492 (9)	
H36A	0.2182	0.3463	0.1833	0.059*	
H36B	0.1912	0.2358	0.1441	0.059*	
C44	0.15538 (13)	0.5070 (3)	0.2676 (2)	0.0488 (9)	
H44	0.1427	0.4490	0.2990	0.059*	
C16	0.33427 (11)	0.2449 (3)	0.5705 (2)	0.0434 (8)	
H16A	0.3324	0.1662	0.5584	0.052*	
H16B	0.3117	0.2637	0.6138	0.052*	
C29	0.41922 (14)	0.0090 (3)	0.2539 (2)	0.0520 (9)	
H29	0.4339	−0.0543	0.2834	0.062*	
C33	0.48036 (13)	0.1160 (3)	0.3656 (2)	0.0466 (8)	
H33A	0.5112	0.1201	0.3410	0.070*	
H33B	0.4807	0.0514	0.4044	0.070*	
H33C	0.4771	0.1804	0.4030	0.070*	
C37	0.21720 (11)	0.3253 (3)	0.0408 (2)	0.0492 (9)	
H37A	0.1941	0.3867	0.0268	0.059*	
H37B	0.2070	0.2668	−0.0047	0.059*	
C50	0.46145 (12)	0.5544 (3)	0.0915 (2)	0.0498 (9)	
H50A	0.4560	0.4818	0.1145	0.075*	
H50B	0.4970	0.5693	0.1008	0.075*	
H50C	0.4476	0.5587	0.0252	0.075*	
C13	0.31123 (13)	0.5060 (3)	0.7362 (2)	0.0546 (9)	
H13	0.2811	0.4689	0.7158	0.065*	
C3	0.37093 (12)	0.8660 (3)	0.4522 (3)	0.0506 (9)	
H3A	0.3893	0.8282	0.4082	0.061*	0.498 (6)
H3B	0.3776	0.8469	0.3891	0.061*	0.502 (6)
C45	0.12403 (14)	0.5668 (3)	0.2040 (3)	0.0587 (10)	
H45	0.0898	0.5502	0.1903	0.070*	
C47	0.19287 (15)	0.6750 (4)	0.1803 (3)	0.0668 (12)	
H47	0.2053	0.7342	0.1500	0.080*	
C52	0.41693 (14)	0.8637 (4)	−0.0232 (3)	0.0722 (13)	
H52A	0.3905	0.8713	−0.0770	0.108*	
H52B	0.4487	0.8563	−0.0443	0.108*	
H52C	0.4177	0.9278	0.0163	0.108*	
C11	0.38662 (15)	0.5233 (4)	0.8454 (3)	0.0675 (12)	
H11	0.4085	0.4988	0.8989	0.081*	
C10	0.39836 (14)	0.6128 (4)	0.7984 (3)	0.0665 (12)	
H10	0.4283	0.6505	0.8195	0.080*	
C12	0.34359 (16)	0.4694 (4)	0.8155 (3)	0.0695 (12)	
H12	0.3354	0.4074	0.8480	0.083*	
C46	0.14300 (16)	0.6512 (4)	0.1605 (3)	0.0794 (14)	
H46	0.1217	0.6935	0.1164	0.095*	
O1B	0.46376 (18)	0.8694 (5)	0.5125 (5)	0.082 (2)	0.502 (6)
C1A	0.4577 (7)	0.8657 (13)	0.6565 (10)	0.065 (4)	0.498 (6)
H1AA	0.4865	0.8781	0.6266	0.098*	0.498 (6)
H1AB	0.4645	0.8052	0.7000	0.098*	0.498 (6)
H1AC	0.4510	0.9308	0.6902	0.098*	0.498 (6)
C4A	0.3372 (3)	0.9666 (6)	0.3947 (6)	0.0338 (16)	0.498 (6)
H4AA	0.314 (3)	1.001 (6)	0.437 (5)	0.051*	0.498 (6)
H4AB	0.318 (3)	0.951 (6)	0.348 (5)	0.051*	0.498 (6)
H4AC	0.365 (3)	1.022 (6)	0.380 (5)	0.051*	0.498 (6)
C2B	0.4233 (4)	0.8525 (9)	0.5327 (8)	0.051 (3)	0.502 (6)
O11	0.2727 (2)	0.8908 (5)	0.1778 (4)	0.0166 (14)	0.25
H11A	0.3048	0.8916	0.1906	0.025*	0.25
H11B	0.2657	0.8381	0.1383	0.025*	0.25
C4B	0.3574 (3)	0.9738 (5)	0.4551 (7)	0.049 (2)	0.502 (6)
H4BA	0.3299	0.9887	0.4044	0.074*	0.502 (6)
H4BB	0.3856	1.0197	0.4483	0.074*	0.502 (6)
H4BC	0.3471	0.9891	0.5146	0.074*	0.502 (6)
C2A	0.4049 (2)	0.9141 (6)	0.5214 (4)	0.0317 (15)	0.498 (6)
O2B	0.4184 (6)	0.8407 (16)	0.6190 (9)	0.044 (3)	0.502 (6)
O1A	0.42431 (16)	1.0015 (3)	0.5223 (3)	0.0492 (16)	0.498 (6)
C1B	0.4595 (6)	0.8377 (14)	0.6925 (11)	0.081 (5)	0.502 (6)
H1BA	0.4832	0.7835	0.6788	0.121*	0.502 (6)
H1BB	0.4483	0.8188	0.7504	0.121*	0.502 (6)
H1BC	0.4754	0.9088	0.6989	0.121*	0.502 (6)

### Atomic displacement parameters (Å^2^)

**Table:**

	*U*^11^	*U*^22^	*U*^33^	*U*^12^	*U*^13^	*U*^23^
O5	0.0269 (9)	0.0271 (10)	0.0227 (9)	0.0019 (8)	−0.0009 (8)	−0.0012 (8)
O4	0.0254 (10)	0.0376 (12)	0.0472 (12)	−0.0104 (9)	0.0069 (8)	−0.0008 (10)
O6	0.0302 (10)	0.0322 (11)	0.0318 (10)	0.0050 (9)	0.0054 (8)	−0.0008 (9)
N3	0.0254 (12)	0.0256 (12)	0.0232 (11)	−0.0044 (9)	0.0002 (9)	0.0021 (9)
N2	0.0221 (11)	0.0308 (13)	0.0348 (13)	−0.0061 (10)	0.0056 (9)	−0.0074 (11)
O7	0.0503 (14)	0.0743 (17)	0.0343 (11)	0.0200 (12)	0.0064 (10)	0.0256 (12)
O3	0.0292 (12)	0.0309 (12)	0.114 (2)	−0.0076 (9)	0.0349 (13)	−0.0243 (13)
O9	0.0327 (12)	0.0701 (18)	0.0595 (15)	0.0071 (11)	0.0126 (11)	0.0410 (13)
N1	0.0259 (12)	0.0284 (13)	0.0554 (16)	−0.0076 (10)	0.0196 (11)	−0.0116 (12)
N5	0.0354 (13)	0.0248 (12)	0.0246 (12)	0.0065 (10)	0.0099 (10)	0.0053 (10)
N4	0.0309 (12)	0.0303 (13)	0.0290 (12)	−0.0028 (10)	0.0040 (10)	−0.0103 (10)
O2A	0.041 (5)	0.038 (5)	0.065 (10)	−0.016 (3)	−0.026 (7)	0.003 (8)
O10	0.0492 (15)	0.0653 (18)	0.0834 (19)	−0.0116 (14)	0.0069 (13)	0.0320 (15)
C19	0.0295 (14)	0.0124 (12)	0.0231 (13)	−0.0013 (10)	0.0014 (11)	−0.0028 (10)
O8	0.0794 (18)	0.0229 (12)	0.110 (2)	−0.0139 (12)	0.0500 (16)	−0.0127 (13)
N6	0.0345 (13)	0.0342 (13)	0.0325 (13)	−0.0013 (11)	0.0081 (10)	0.0046 (11)
C20	0.0277 (13)	0.0161 (12)	0.0222 (13)	0.0028 (10)	0.0005 (10)	0.0058 (11)
C14	0.0282 (15)	0.0322 (15)	0.0219 (14)	−0.0075 (12)	0.0020 (11)	0.0003 (12)
C22	0.0305 (15)	0.0233 (15)	0.0342 (15)	−0.0039 (12)	0.0000 (12)	0.0053 (12)
C21	0.0305 (15)	0.0200 (13)	0.0269 (14)	0.0013 (11)	0.0000 (11)	0.0012 (11)
C39	0.0246 (14)	0.052 (2)	0.0257 (15)	0.0092 (13)	0.0095 (12)	0.0095 (14)
C34	0.0337 (15)	0.0254 (14)	0.0272 (14)	0.0013 (12)	0.0019 (12)	−0.0103 (12)
C25	0.0307 (14)	0.0229 (14)	0.0241 (13)	0.0047 (11)	0.0006 (11)	0.0036 (11)
C15	0.0274 (14)	0.0286 (15)	0.0300 (15)	−0.0091 (11)	0.0020 (11)	0.0045 (12)
C23	0.0269 (14)	0.0369 (17)	0.0379 (17)	−0.0005 (13)	0.0057 (12)	0.0097 (14)
C32	0.0427 (17)	0.0248 (15)	0.0337 (16)	0.0039 (13)	0.0028 (13)	−0.0065 (12)
C5	0.0258 (15)	0.0318 (17)	0.054 (2)	−0.0077 (12)	0.0160 (14)	−0.0184 (14)
C42	0.0492 (18)	0.0316 (16)	0.0422 (17)	0.0097 (14)	0.0227 (14)	−0.0042 (14)
C8	0.0328 (15)	0.0426 (18)	0.0371 (16)	0.0007 (13)	0.0116 (13)	−0.0186 (15)
C18	0.0313 (15)	0.0407 (17)	0.0315 (15)	−0.0012 (13)	−0.0032 (12)	0.0135 (14)
C24	0.0320 (15)	0.0343 (17)	0.0319 (15)	0.0077 (13)	0.0070 (12)	0.0033 (13)
C6	0.0210 (13)	0.0302 (15)	0.0406 (16)	−0.0080 (12)	0.0084 (11)	−0.0110 (13)
C28	0.0498 (18)	0.0244 (15)	0.0389 (17)	0.0049 (14)	0.0033 (14)	−0.0024 (14)
C38	0.0255 (14)	0.057 (2)	0.0208 (13)	0.0045 (14)	0.0019 (11)	−0.0098 (14)
C27	0.0378 (16)	0.0226 (14)	0.0316 (15)	0.0033 (12)	0.0069 (12)	−0.0036 (12)
C7	0.0213 (14)	0.043 (2)	0.0457 (18)	−0.0077 (13)	0.0109 (13)	−0.0152 (15)
C40	0.0463 (17)	0.0194 (15)	0.0407 (16)	0.0077 (12)	0.0168 (13)	0.0088 (12)
C48	0.055 (2)	0.0228 (16)	0.0453 (18)	0.0042 (14)	0.0211 (15)	0.0123 (14)
C51	0.0326 (17)	0.057 (2)	0.051 (2)	0.0011 (16)	0.0109 (15)	0.0229 (17)
C41	0.058 (2)	0.0239 (16)	0.0442 (18)	0.0049 (14)	0.0228 (15)	0.0003 (13)
C49	0.0352 (16)	0.0478 (19)	0.0303 (15)	−0.0007 (14)	0.0030 (12)	0.0116 (14)
C31	0.059 (2)	0.0280 (18)	0.0443 (19)	0.0017 (15)	−0.0071 (15)	−0.0127 (14)
C17	0.0407 (17)	0.065 (2)	0.0304 (16)	−0.0062 (16)	−0.0007 (13)	0.0203 (16)
C43	0.056 (2)	0.0351 (18)	0.0337 (16)	0.0022 (15)	0.0160 (14)	−0.0066 (14)
C26	0.0419 (18)	0.052 (2)	0.0469 (19)	0.0102 (16)	0.0174 (15)	−0.0010 (16)
C35	0.0485 (19)	0.0351 (17)	0.0394 (17)	−0.0185 (15)	0.0138 (14)	−0.0148 (14)
C9	0.0289 (17)	0.046 (2)	0.075 (2)	0.0047 (14)	−0.0034 (16)	−0.0197 (18)
C30	0.079 (3)	0.0222 (17)	0.060 (2)	0.0023 (17)	−0.0101 (19)	−0.0131 (16)
C36	0.0418 (18)	0.049 (2)	0.062 (2)	−0.0186 (16)	0.0242 (16)	−0.0270 (18)
C44	0.060 (2)	0.042 (2)	0.051 (2)	−0.0074 (17)	0.0283 (17)	−0.0189 (17)
C16	0.0408 (18)	0.051 (2)	0.0391 (18)	−0.0072 (15)	0.0079 (14)	0.0166 (15)
C29	0.071 (2)	0.0218 (16)	0.058 (2)	0.0099 (16)	−0.0032 (18)	−0.0010 (16)
C33	0.057 (2)	0.0244 (16)	0.052 (2)	0.0060 (15)	−0.0065 (16)	0.0037 (15)
C37	0.0285 (16)	0.066 (2)	0.052 (2)	−0.0036 (15)	0.0053 (14)	−0.0270 (18)
C50	0.0316 (17)	0.073 (2)	0.0423 (19)	0.0083 (17)	0.0006 (14)	0.0043 (18)
C13	0.055 (2)	0.075 (3)	0.0339 (18)	−0.022 (2)	0.0101 (15)	−0.0034 (18)
C3	0.048 (2)	0.0403 (19)	0.073 (2)	−0.0194 (16)	0.0389 (18)	−0.0190 (18)
C45	0.047 (2)	0.069 (3)	0.063 (2)	0.009 (2)	0.0205 (19)	−0.013 (2)
C47	0.060 (2)	0.064 (3)	0.085 (3)	0.023 (2)	0.035 (2)	0.034 (2)
C52	0.047 (2)	0.088 (3)	0.089 (3)	0.017 (2)	0.032 (2)	0.066 (3)
C11	0.059 (3)	0.093 (4)	0.046 (2)	0.008 (2)	−0.0040 (18)	−0.026 (2)
C10	0.042 (2)	0.062 (3)	0.087 (3)	0.0087 (19)	−0.013 (2)	−0.031 (2)
C12	0.079 (3)	0.091 (3)	0.039 (2)	−0.013 (2)	0.013 (2)	0.005 (2)
C46	0.053 (3)	0.095 (4)	0.094 (3)	0.031 (2)	0.022 (2)	0.029 (3)
O1B	0.034 (3)	0.090 (5)	0.129 (6)	−0.012 (3)	0.029 (3)	0.011 (4)
C1A	0.067 (8)	0.054 (7)	0.063 (8)	−0.011 (6)	−0.020 (6)	0.018 (5)
C4A	0.029 (4)	0.035 (4)	0.038 (4)	0.007 (3)	0.007 (3)	0.009 (3)
C2B	0.040 (5)	0.023 (5)	0.095 (9)	−0.004 (4)	0.027 (6)	−0.008 (5)
O11	0.016 (3)	0.015 (3)	0.019 (3)	−0.002 (3)	0.005 (3)	−0.001 (3)
C4B	0.051 (5)	0.035 (4)	0.069 (6)	−0.002 (3)	0.028 (5)	0.012 (4)
C2A	0.024 (3)	0.025 (4)	0.048 (4)	0.000 (3)	0.012 (3)	0.001 (3)
O2B	0.023 (4)	0.034 (4)	0.065 (8)	−0.008 (3)	−0.017 (6)	−0.012 (6)
O1A	0.041 (3)	0.027 (3)	0.074 (3)	−0.017 (2)	−0.006 (2)	0.009 (2)
C1B	0.039 (5)	0.087 (11)	0.101 (12)	−0.018 (6)	−0.031 (7)	−0.012 (9)

### Geometric parameters (Å, º)

**Table:**

O5—C19	1.239 (3)	C49—C50	1.517 (5)
O4—C14	1.235 (3)	C31—H31	0.9500
O6—C34	1.239 (3)	C31—C30	1.374 (5)
N3—C19	1.343 (3)	C17—H17A	0.9900
N3—C15	1.466 (3)	C17—H17B	0.9900
N3—C18	1.486 (3)	C17—C16	1.530 (4)
N2—H2	0.8800	C43—H43	0.9500
N2—C14	1.328 (3)	C43—C44	1.388 (5)
N2—C6	1.449 (4)	C26—H26A	0.9800
O7—C39	1.228 (4)	C26—H26B	0.9800
O3—C5	1.232 (3)	C26—H26C	0.9800
O9—C51	1.330 (4)	C35—H35A	0.9900
O9—C52	1.452 (4)	C35—H35B	0.9900
N1—H1	0.8800	C35—C36	1.521 (5)
N1—C5	1.329 (4)	C9—H9	0.9500
N1—C3	1.461 (4)	C9—C10	1.369 (5)
N5—H5	0.8800	C30—H30	0.9500
N5—C39	1.341 (4)	C30—C29	1.371 (5)
N5—C40	1.450 (4)	C36—H36A	0.9900
N4—C34	1.355 (3)	C36—H36B	0.9900
N4—C38	1.459 (4)	C36—C37	1.526 (5)
N4—C35	1.486 (4)	C44—H44	0.9500
O2A—C1A	1.449 (14)	C44—C45	1.371 (5)
O2A—C2A	1.314 (15)	C16—H16A	0.9900
O10—C51	1.203 (4)	C16—H16B	0.9900
C19—C20	1.501 (4)	C29—H29	0.9500
O8—C48	1.225 (4)	C33—H33A	0.9800
N6—H6	0.8800	C33—H33B	0.9800
N6—C48	1.338 (4)	C33—H33C	0.9800
N6—C49	1.452 (4)	C37—H37A	0.9900
C20—C21	1.397 (4)	C37—H37B	0.9900
C20—C25	1.401 (4)	C50—H50A	0.9800
C14—C15	1.524 (4)	C50—H50B	0.9800
C22—H22	0.9500	C50—H50C	0.9800
C22—C21	1.375 (4)	C13—H13	0.9500
C22—C23	1.387 (4)	C13—C12	1.409 (5)
C21—H21	0.9500	C3—H3A	1.0000
C39—C38	1.513 (5)	C3—H3B	1.0000
C34—C32	1.498 (4)	C3—C4A	1.688 (8)
C25—C24	1.404 (4)	C3—C2B	1.704 (13)
C25—C27	1.510 (4)	C3—C4B	1.389 (7)
C15—H15	1.0000	C3—C2A	1.388 (7)
C15—C16	1.529 (4)	C45—H45	0.9500
C23—H23	0.9500	C45—C46	1.373 (6)
C23—C24	1.388 (4)	C47—H47	0.9500
C32—C27	1.402 (4)	C47—C46	1.384 (6)
C32—C31	1.396 (4)	C52—H52A	0.9800
C5—C6	1.540 (4)	C52—H52B	0.9800
C42—C41	1.509 (5)	C52—H52C	0.9800
C42—C43	1.379 (4)	C11—H11	0.9500
C42—C47	1.377 (5)	C11—C10	1.372 (6)
C8—C7	1.513 (4)	C11—C12	1.363 (6)
C8—C9	1.389 (4)	C10—H10	0.9500
C8—C13	1.363 (5)	C12—H12	0.9500
C18—H18A	0.9900	C46—H46	0.9500
C18—H18B	0.9900	O1B—C2B	1.218 (10)
C18—C17	1.526 (4)	C1A—H1AA	0.9800
C24—C26	1.517 (4)	C1A—H1AB	0.9800
C6—H6A	1.0000	C1A—H1AC	0.9800
C6—C7	1.537 (4)	C4A—H4AA	1.05 (7)
C28—C27	1.403 (4)	C4A—H4AB	0.81 (8)
C28—C29	1.395 (5)	C4A—H4AC	1.08 (8)
C28—C33	1.517 (4)	C2B—O2B	1.301 (17)
C38—H38	1.0000	O11—H11A	0.8705
C38—C37	1.537 (4)	O11—H11B	0.8699
C7—H7A	0.99 (4)	C4B—H4BA	0.9800
C7—H7B	0.96 (3)	C4B—H4BB	0.9800
C40—H40	1.0000	C4B—H4BC	0.9800
C40—C48	1.519 (4)	C2A—O1A	1.205 (8)
C40—C41	1.525 (4)	O2B—C1B	1.419 (13)
C51—C49	1.518 (5)	C1B—H1BA	0.9800
C41—H41A	0.9900	C1B—H1BB	0.9800
C41—H41B	0.9900	C1B—H1BC	0.9800
C49—H49	1.0000		
			
C19—N3—C15	119.8 (2)	C16—C17—H17B	111.2
C19—N3—C18	127.6 (2)	C42—C43—H43	119.4
C15—N3—C18	111.8 (2)	C42—C43—C44	121.1 (3)
C14—N2—H2	116.9	C44—C43—H43	119.4
C14—N2—C6	126.3 (2)	C24—C26—H26A	109.5
C6—N2—H2	116.9	C24—C26—H26B	109.5
C51—O9—C52	114.9 (3)	C24—C26—H26C	109.5
C5—N1—H1	119.5	H26A—C26—H26B	109.5
C5—N1—C3	121.0 (2)	H26A—C26—H26C	109.5
C3—N1—H1	119.5	H26B—C26—H26C	109.5
C39—N5—H5	119.1	N4—C35—H35A	111.2
C39—N5—C40	121.9 (2)	N4—C35—H35B	111.2
C40—N5—H5	119.1	N4—C35—C36	102.8 (3)
C34—N4—C38	120.9 (2)	H35A—C35—H35B	109.1
C34—N4—C35	127.2 (3)	C36—C35—H35A	111.2
C38—N4—C35	112.0 (2)	C36—C35—H35B	111.2
C2A—O2A—C1A	114.1 (14)	C8—C9—H9	119.3
O5—C19—N3	120.5 (2)	C10—C9—C8	121.4 (4)
O5—C19—C20	120.8 (2)	C10—C9—H9	119.3
N3—C19—C20	118.5 (2)	C31—C30—H30	119.9
C48—N6—H6	119.3	C29—C30—C31	120.2 (3)
C48—N6—C49	121.5 (3)	C29—C30—H30	119.9
C49—N6—H6	119.3	C35—C36—H36A	111.1
C21—C20—C19	117.9 (2)	C35—C36—H36B	111.1
C21—C20—C25	120.6 (2)	C35—C36—C37	103.1 (2)
C25—C20—C19	121.3 (2)	H36A—C36—H36B	109.1
O4—C14—N2	123.2 (3)	C37—C36—H36A	111.1
O4—C14—C15	120.1 (2)	C37—C36—H36B	111.1
N2—C14—C15	116.7 (2)	C43—C44—H44	119.7
C21—C22—H22	120.3	C45—C44—C43	120.6 (3)
C21—C22—C23	119.4 (3)	C45—C44—H44	119.7
C23—C22—H22	120.3	C15—C16—C17	103.5 (2)
C20—C21—H21	119.9	C15—C16—H16A	111.1
C22—C21—C20	120.1 (3)	C15—C16—H16B	111.1
C22—C21—H21	119.9	C17—C16—H16A	111.1
O7—C39—N5	123.8 (3)	C17—C16—H16B	111.1
O7—C39—C38	119.0 (3)	H16A—C16—H16B	109.0
N5—C39—C38	117.2 (2)	C28—C29—H29	119.4
O6—C34—N4	121.7 (3)	C30—C29—C28	121.2 (3)
O6—C34—C32	121.8 (2)	C30—C29—H29	119.4
N4—C34—C32	116.3 (3)	C28—C33—H33A	109.5
C20—C25—C24	118.9 (2)	C28—C33—H33B	109.5
C20—C25—C27	122.3 (2)	C28—C33—H33C	109.5
C24—C25—C27	118.3 (2)	H33A—C33—H33B	109.5
N3—C15—C14	113.6 (2)	H33A—C33—H33C	109.5
N3—C15—H15	109.1	H33B—C33—H33C	109.5
N3—C15—C16	103.9 (2)	C38—C37—H37A	111.1
C14—C15—H15	109.1	C38—C37—H37B	111.1
C14—C15—C16	111.9 (2)	C36—C37—C38	103.3 (2)
C16—C15—H15	109.1	C36—C37—H37A	111.1
C22—C23—H23	119.2	C36—C37—H37B	111.1
C22—C23—C24	121.7 (3)	H37A—C37—H37B	109.1
C24—C23—H23	119.2	C49—C50—H50A	109.5
C27—C32—C34	123.4 (2)	C49—C50—H50B	109.5
C31—C32—C34	116.5 (3)	C49—C50—H50C	109.5
C31—C32—C27	120.0 (3)	H50A—C50—H50B	109.5
O3—C5—N1	123.2 (3)	H50A—C50—H50C	109.5
O3—C5—C6	120.2 (3)	H50B—C50—H50C	109.5
N1—C5—C6	116.6 (2)	C8—C13—H13	119.7
C43—C42—C41	121.6 (3)	C8—C13—C12	120.5 (3)
C47—C42—C41	120.7 (3)	C12—C13—H13	119.7
C47—C42—C43	117.7 (3)	N1—C3—H3A	108.7
C9—C8—C7	120.8 (3)	N1—C3—H3B	110.4
C13—C8—C7	120.8 (3)	N1—C3—C4A	106.0 (3)
C13—C8—C9	118.3 (3)	N1—C3—C2B	105.5 (4)
N3—C18—H18A	111.3	C4A—C3—H3A	108.7
N3—C18—H18B	111.3	C2B—C3—H3B	110.4
N3—C18—C17	102.3 (2)	C4B—C3—N1	114.2 (4)
H18A—C18—H18B	109.2	C4B—C3—H3B	110.4
C17—C18—H18A	111.3	C4B—C3—C2B	105.8 (6)
C17—C18—H18B	111.3	C2A—C3—N1	117.8 (4)
C25—C24—C26	120.6 (3)	C2A—C3—H3A	108.7
C23—C24—C25	119.2 (3)	C2A—C3—C4A	106.8 (5)
C23—C24—C26	120.2 (3)	C44—C45—H45	120.6
N2—C6—C5	104.6 (2)	C44—C45—C46	118.8 (4)
N2—C6—H6A	107.9	C46—C45—H45	120.6
N2—C6—C7	113.9 (3)	C42—C47—H47	119.3
C5—C6—H6A	107.9	C42—C47—C46	121.3 (4)
C7—C6—C5	114.3 (2)	C46—C47—H47	119.3
C7—C6—H6A	107.9	O9—C52—H52A	109.5
C27—C28—C33	121.9 (3)	O9—C52—H52B	109.5
C29—C28—C27	119.1 (3)	O9—C52—H52C	109.5
C29—C28—C33	119.0 (3)	H52A—C52—H52B	109.5
N4—C38—C39	115.6 (2)	H52A—C52—H52C	109.5
N4—C38—H38	109.3	H52B—C52—H52C	109.5
N4—C38—C37	103.5 (3)	C10—C11—H11	120.0
C39—C38—H38	109.3	C12—C11—H11	120.0
C39—C38—C37	109.6 (2)	C12—C11—C10	120.1 (4)
C37—C38—H38	109.3	C9—C10—C11	119.9 (4)
C32—C27—C25	123.8 (2)	C9—C10—H10	120.1
C32—C27—C28	119.2 (3)	C11—C10—H10	120.1
C28—C27—C25	116.9 (2)	C13—C12—H12	120.1
C8—C7—C6	114.8 (2)	C11—C12—C13	119.8 (4)
C8—C7—H7A	110.1 (19)	C11—C12—H12	120.1
C8—C7—H7B	107.6 (19)	C45—C46—C47	120.5 (4)
C6—C7—H7A	108.4 (19)	C45—C46—H46	119.7
C6—C7—H7B	109.5 (19)	C47—C46—H46	119.7
H7A—C7—H7B	106 (3)	O2A—C1A—H1AA	109.5
N5—C40—H40	106.6	O2A—C1A—H1AB	109.5
N5—C40—C48	113.0 (2)	O2A—C1A—H1AC	109.5
N5—C40—C41	110.3 (2)	H1AA—C1A—H1AB	109.5
C48—C40—H40	106.6	H1AA—C1A—H1AC	109.5
C48—C40—C41	113.2 (3)	H1AB—C1A—H1AC	109.5
C41—C40—H40	106.6	C3—C4A—H4AA	110 (4)
O8—C48—N6	122.2 (3)	C3—C4A—H4AB	117 (5)
O8—C48—C40	121.5 (3)	C3—C4A—H4AC	102 (4)
N6—C48—C40	116.2 (3)	H4AA—C4A—H4AB	102 (6)
O9—C51—C49	112.6 (3)	H4AA—C4A—H4AC	113 (5)
O10—C51—O9	124.8 (3)	H4AB—C4A—H4AC	112 (7)
O10—C51—C49	122.3 (3)	O1B—C2B—C3	120.9 (9)
C42—C41—C40	111.3 (3)	O1B—C2B—O2B	120.6 (13)
C42—C41—H41A	109.4	O2B—C2B—C3	117.7 (9)
C42—C41—H41B	109.4	H11A—O11—H11B	104.4
C40—C41—H41A	109.4	C3—C4B—H4BA	109.5
C40—C41—H41B	109.4	C3—C4B—H4BB	109.5
H41A—C41—H41B	108.0	C3—C4B—H4BC	109.5
N6—C49—C51	114.6 (2)	H4BA—C4B—H4BB	109.5
N6—C49—H49	108.6	H4BA—C4B—H4BC	109.5
N6—C49—C50	108.9 (3)	H4BB—C4B—H4BC	109.5
C51—C49—H49	108.6	O2A—C2A—C3	105.3 (9)
C50—C49—C51	107.5 (3)	O1A—C2A—O2A	125.2 (10)
C50—C49—H49	108.6	O1A—C2A—C3	129.3 (6)
C32—C31—H31	119.9	C2B—O2B—C1B	122.3 (15)
C30—C31—C32	120.2 (3)	O2B—C1B—H1BA	109.5
C30—C31—H31	119.9	O2B—C1B—H1BB	109.5
C18—C17—H17A	111.2	O2B—C1B—H1BC	109.5
C18—C17—H17B	111.2	H1BA—C1B—H1BB	109.5
C18—C17—C16	103.0 (2)	H1BA—C1B—H1BC	109.5
H17A—C17—H17B	109.1	H1BB—C1B—H1BC	109.5
C16—C17—H17A	111.2		
			
C20—C19—N3—C15	178.3 (2)	C32—C34—N4—C38	−164.6 (2)
C19—N3—C15—C14	−73.4 (3)	C34—N4—C38—C39	−69.1 (3)
N3—C15—C14—N2	−17.5 (3)	N4—C38—C39—N5	−14.4 (4)
C15—C14—N2—C6	176.5 (2)	C38—C39—N5—C40	−177.2 (2)
C14—N2—C6—C5	−163.0 (2)	C39—N5—C40—C48	−106.8 (3)
N2—C6—C5—N1	171.4 (2)	N5—C40—C48—N6	18.6 (3)
C6—C5—N1—C3	−174.8 (3)	C40—C48—N6—C49	179.1 (2)
C5—N1—C3—C2B	−58.0 (5)	C48—N6—C49—C51	−60.9 (3)
N1—C3—C2B—O2B	−39.6 (13)	N6—C49—C51—O9	−35.0 (4)

### Hydrogen-bond geometry (Å, º)

*Cg*3 and *Cg*5 are the centroids of the C8–C13 and C27–C32 rings, respectively.

**Table:**

*D*—H···*A*	*D*—H	H···*A*	*D*···*A*	*D*—H···*A*
N5—H5···O5	0.88	2.07	2.923 (3)	162
N6—H6···O6	0.88	2.42	3.233 (3)	154
C9—H9···O2*B*	0.95	2.35	3.270 (18)	164
C21—H21···O3	0.95	2.44	3.352 (4)	161
C35—H35*A*···O5	0.99	2.51	3.171 (4)	124
C43—H43···O4	0.95	2.59	3.443 (4)	149
N1—H1···O4^i^	0.88	2.01	2.865 (3)	163
C1*B*—H1*BB*···O10^ii^	0.98	2.46	2.913 (16)	108
C30—H30···O8^iii^	0.95	2.46	3.222 (4)	137
C35—H35···O7^iv^	0.99	2.39	3.228 (4)	142
C52—H52*B*···O10^v^	0.98	2.60	3.559 (5)	166
O11—H11*A*···O8	0.87	2.48	3.136 (6)	133
C13—H13···O11^vi^	0.95	2.52	3.155 (7)	124
C36—H36*B*···*Cg*3^vi^	0.99	2.94	3.845 (4)	152
C4*A*—H4*AC*···*Cg*5^vii^	1.05 (8)	2.93 (7)	3.770 (8)	135 (5)

Symmetry codes: (i) −*x*+1/2, *y*+1/2, −*z*+1; (ii) −*x*+1, *y*, −*z*+1; (iii) *x*, *y*−1, *z*; (iv) −*x*+1/2, *y*−1/2, −*z*; (v) −*x*+1, *y*, −*z*; (vi) −*x*+1/2, *y*−1/2, −*z*+1; (vii) *x*, *y*+1, *z*.
