# Supplementary material for: Synthesis and crystal structure of peptide dimethyl biphenyl hybrid C52H60N6O10·0.25H2O
Source: Acta Crystallogr E Crystallogr Commun. 2020 Sep 25;76(Pt 10):1675–8. doi: 10.1107/S2056989020012931 (PMC7534232; doi:10.1107/S2056989020012931)
Supplement: Supplementary file 4 [file e-76-01675-sup5.docx]

**Synthesis and crystal structure of peptide di­methyl bi­phenyl hybrid C_52_H_60_N_6_O_10_·0.25H_2_O**

Xuan Tu Nguyen, Thuy Quynh Le, Tra My Bui Thi, Dinh Hung Mac and Thai Thanh Thu Bui*

Department of Chemistry, VNU University of science, Vietnam National University, Hanoi, 19 Le Thanh Tong, Hanoi, Vietnam

Correspondence email: thaithanhthubui@gmail.com

The crystal structure of title compound shows a disorder of methyl and carboxylic group of Alanine amino acid. The torsion angles of amino acids residues are difference compared with the angles of peptide biphenyl hybrids previously reported.

Abstract

The synthesis and crystal structure of peptide 6,6’-dimethyl biphenyl hybrid are described. The title compound was synthetized by reaction between 6,6'-dimethyl-[1,1'-biphenyl]-2,2'-dicarbonyl dichloride in CH2Cl2, amine and Et3N at 273K under N2 atmosphere. Its crystal was characterized by single crystal X ray diffraction method. The crystallographic analysis provided an understanding of architecture and structural of crystal. In the crystal, one asymmetric unit contain one molecule of this peptide and a quarter of a water molecule. The structure is stabilized by some hydrogen bond intra- and inter-molecular.

Keywords: crystal structure; hydrogen bonding, peptide dimethyl biphenyl hybrids

CCDC reference: 2026794

1. Chemical context

Since the first application of peptide in treatment of diabetes with insulin from 1922 (Banting et al., 1922), the chemistry of peptide has become a very important domain in the search of new therapeutic drug. From 2011 to 2018, the global market of drug has increased from 14.1 to 24.4 billion US dollar. With more than 140 peptides in clinical trials the number of peptide-based drug is expected to grow significantly (Fosgerau et al., 2015). Despite its tremendous potential, applications of peptides for pharmaceutical purposes are limited by their instability toward enzymatic system, short half-life, rapid renal clearance, and formulation challenges (Otvos et al., 2014). These problems can be overcome by modifying the linear peptide to enhance the stability and therefore the selectivity and affinity. The biphenyl structure is present in numerous pharmaceuticals and bioactive compound as illustrated by the glycopeptide antibiotic vancomycin, the proteasome inhibitor TMC-95A (Kaiser et al., 2004), arylomycins (Schimana et al., 2002). A statistical analysis of NMR indicates that compound containing the biphenyl structure can bind a wide range of proteins with high levels of specificity (Hajduk et al., 2000). Coupling of a small protein chain to the biphenyl structure is strategy to create new family of peptidomimetic compounds which can be used in medicinal chemistry because of its specific conformation and its particular hydrogen bonding interaction.

| ****  **A** | ****  **B** |
| --- | --- |

Figure 1. Peptide-biphenyl hybrids A & B

The synthesis and biological activity as calpain inhibitor of peptide-biphenyl hybrids type I have been reported by Montero and Mann (Montero et al., 2004a & 2004b; Mann et al., 2002). Amine synthesized a bis amido-copper (II) complex from N-containing tetradentate ligands having two amido groups with a biphenyl skeleton which is used as an DNA cleaving agent (Amine et al., 2002). Recently, we have reported crystallographic studies of a peptide-biphenyl hybrids **A** (figure 1 A) with tripeptide Pro-Phe-Ala (Le et al., 2020).

We report herein the synthesis and crystallographic study of a peptide-2,2’-biphenyl **B** (figure 1B) with introduction of two methyl group at 6-6’ position to prevent free rotation around the aryl-aryl bond.

Scheme 1. Structure of title compound

1. Structure commentary

The compound dimethyl 2,2'-(((2S,2'S)-2,2'-(((2S,2'S)-1,1'-(6,6'-dimethyl-[1,1'-biphenyl]-2,2'-dicarbonyl)bis(pyrrolidine-1,2-diyl-2-carbonyl))bis(azanediyl))bis(3-phenylpropanoyl))bis(azanediyl))(2S,2'S)-dipropionate crystallizes in monoclinic system in space group C2 with one molecule of peptide biphenyl hybrid accompanied a quarter of a water molecule in one asymmetric unit. Two methyl group have been introduced to biphenyl rings at 6,6’ position in order to limits the rotation of two phenyl rings, in the liquid state. However, in the solid state the dihedral angle between biphenyl rings (C20 – C25) and (C27 – C32) is 73.8(3)^o^. This value is similar with that of previous compound (C_50_H_56_N_6_O_10_.0.5H_2_O, Le et al., 2020). A disorder of methyl and carboxylic group of Alanine amino acid residue occurs in the crystal structure with occupancy ratio 0.502(6) : 0.498(6).


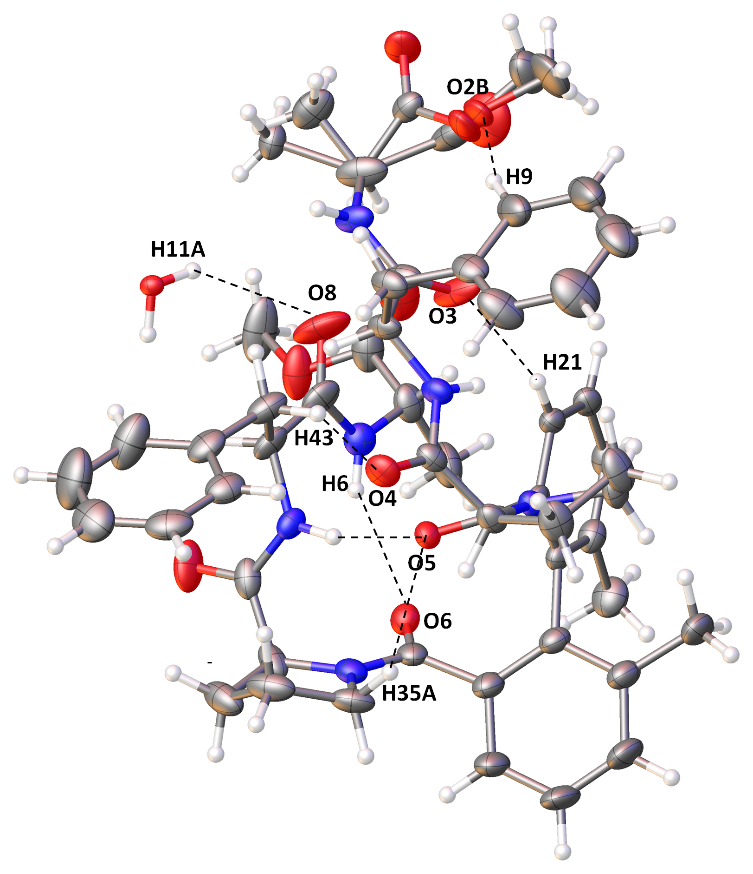


Figure 2. A view of molecular structure of title compound with displacement ellipsoids draw at the 50% probability level and hydrogel bonds (dashed lines) within the asymmetric unit. H atoms are shown as small circles of arbitrary radii.

The backbone conformation of two tripeptide fragment is characterized by their torsion angles ω, φ, ψ (see table 1). The torsion angles φ and ψ of amino acids Ala1, Ala2, Phe2 correspond with α-helix (right-handed) of Ramachandran plot for the general case, and only the relate torsion angles of amino acid Phe1 fall into the corresponding type β-sheet Ramachandran plot region. For Proline amino acid, the relate torsion angles of both Pro lie in α region of Ramachandran plot for Proline.

There are six intramolecular hydrogen bonds formed in the structure of title compound. In which, two bonds are formed between the NH and CO group with the short distance, d = 2.07Å for C5 – N5 ···O5 and d = 2.42 Å for N6 – H6···O6. The last value is quite longer than the value observed (from 2.04 to 2.29 Å) in reported peptides (Ranganathan et al., 1997 & Le et al., 2020). Four other intra bonds are formed between CH and CO group with the distance from 2.35 to 2.59 Å (Table 2).

Table 1. backbone torsion angles ω, φ, ψ (^o^) for the two tripeptide fragments.

| Pro1 | ω1 | C20 – C19 – N3 – C15 | 178.3(2) |
| --- | --- | --- | --- |
|  | φ1 | C19 – N3 – C15 – C14 | -73.4(3) |
|  | ψ1 | N3 – C15 – C14 – N2 | -17.5(3) |
| Phe1 | ω2 | C15 – C14 – N2 – C6 | 176.5(2) |
|  | φ2 | C14 – N2 – C6 – C5 | -163.0(2) |
|  | ψ2 | N2 – C6 – C5 – N1 | 171.4(2) |
| Ala1 | ω3 | C6 – C5 – N1 – C3 | -174.8(3) |
|  | φ3B | C5 – N1 – C3 – C2B | -58.0(5) |
|  | ψ3B | N1 – C3 – C2B – O2B | -39.6(13) |
| Pro2 | ω4 | C32 – C34 – N4 – C38 | -164.6(2) |
|  | φ4 | C34 – N4 – C38 – C39 | -69.1(3) |
|  | ψ4 | N4 – C38 – C39 – N5 | -14.4(4) |
| Phe2 | ω5 | C38 – C39 – N5 – C40 | -177.2(2) |
|  | φ5 | C39 – N5 – C40 – C48 | -106.8(3) |
|  | ψ5 | N5 – C40 – C48 – N6 | 18.6(3) |
| Ala2 | ω6 | C40 – C48 – N6 – C49 | 179.1(2) |
|  | φ6 | C48 – N6 – C49 – C51 | -60.9(3) |
|  | ψ6 | N6 – C49 – C51 – O9 | -35.0(4) |

1. Supramolecular feature

Table 2. Hydrogen bond geometry (Å, ^o^). *Cg*3, *Cg*5 are centroids of C8 – C13 and C27 – C32 rings, respectively.

| D – H ···A | D – H | H ···A | D ···A | D – H ···A |
| --- | --- | --- | --- | --- |
| N5 – H5···O5 | 0.88 | 2.07 | 2.923(3) | 162 |
| N6 – H6···O6 | 0.88 | 2.42 | 3.233(3) | 154 |
| C9 – H9···O2B | 0.95 | 2.35 | 3.270(18) | 164 |
| C21 – H21···O3 | 0.95 | 2.44 | 3.352(4) | 161 |
| C35 – H35A···O5 | 0.99 | 2.51 | 3.171(4) | 124 |
| C43 – H43···O4 | 0.95 | 2.59 | 3.443(4) | 149 |
| N1 – H1···O4^i^ | 0.88 | 2.01 | 2.865(3) | 163 |
| C1B – H1BB···O10^ii^ | 0.98 | 2.46 | 2.913(16) | 108 |
| C30 – H30···O8^iii^ | 0.95 | 2.46 | 3.222(4) | 137 |
| C35 – H35B···O7^iv^ | 0.99 | 2.39 | 3.228(4) | 142 |
| C52 – H52B···O10^v^ | 0.98 | 2.60 | 3.559(5) | 166 |
| O11 – H11A···O8 | 0.87 | 2.48 | 3.136(6) | 133 |
| C13 – H13···O11^vi^ | 0.95 | 2.52 | 3.155(7) | 124 |
| C36 – H36B ··· *Cg*3^vi^ | 0.99 | 2.94 | 3.845(4) | 152 |
| C4A – H4AC ··· *Cg*5^vii^ | 1.08 | 2.93 | 3.770(8) | 135 |

Symmetry code (i) 1/2-*x*,1/2+*y*,1-*z* ; (ii) 1-*x*,*y*,1-*z* ; (iii) *x*,-1+*y*,*z* ; (iv) 1/2-*x*,-1/2+*y*,-*z* ; (v) 1-*x*,*y*,-*z* ; (vi) ½-*x*, -1/2+*y*, 1-*z*;  (vii) *x*, 1+*y*, *z*

In the crystal, the packing is directed by hydrogen bonding (see table 2). An intermolecular interaction very strong is formed between NH and CO group of peptide residues (N1 – H1···O4^i^, with d = 2.01 Å, symmetry code i: 1/2-*x*,1/2+*y*,1-*z*). Aside from that interaction, there are 6 hydrogen bonding link the molecules. Therein, two contact are established from water molecule with two tripeptides (O11 – H11A···O8; C13 – H13···O11). Four C – H ···O = C contacts with H···O distances ranging from 2.39 to 2.60 Å are also observed. In addition, the molecules are linked by two intermolecular C – H ···π interactions.


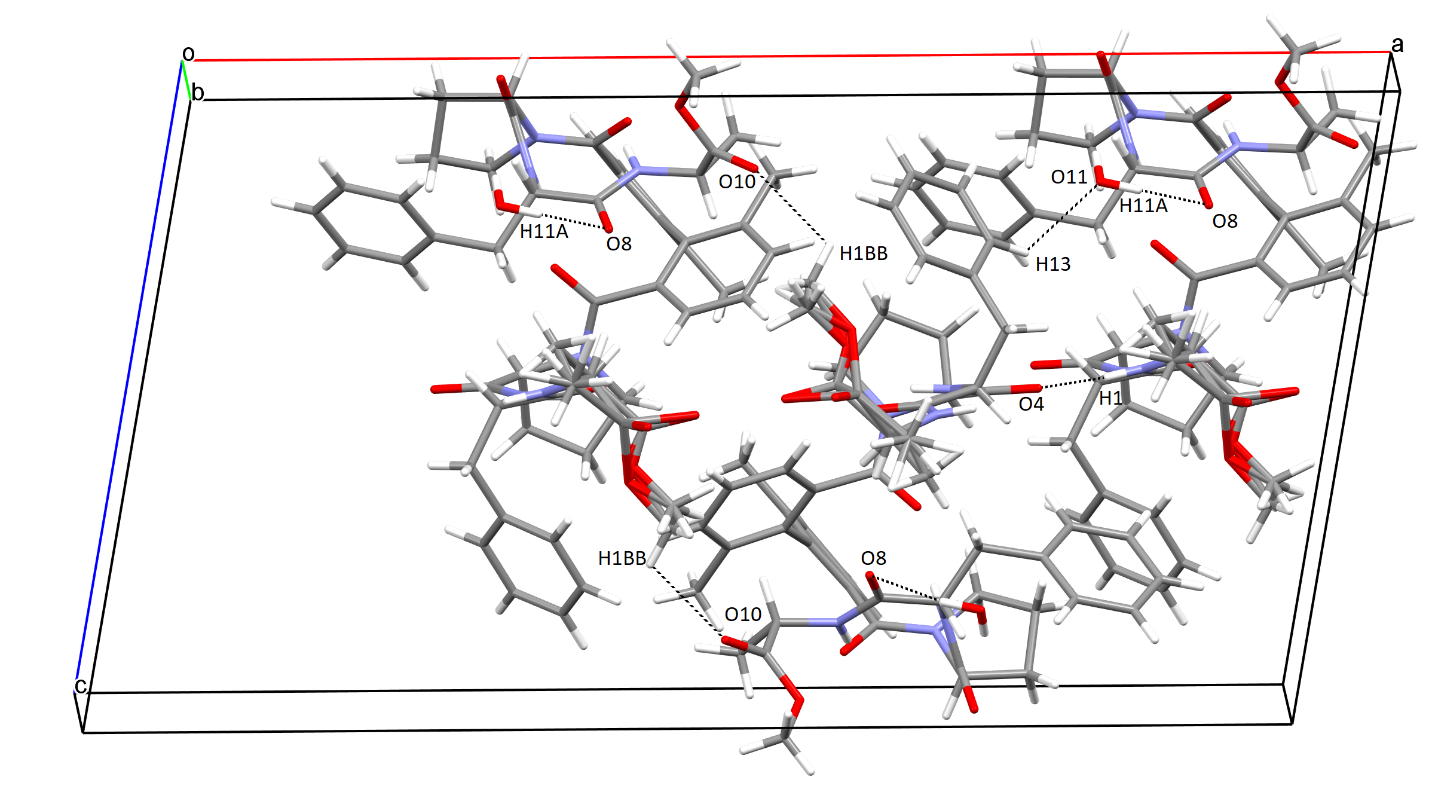


Figure 3. Crystal packing of title compound, indicating some hydrogel bonding intermolecular (dashed line).

1. Database survey

A search of the Cambridge Structure Database (5.41 version with March 2020 update, Groom et al., 2016) for peptide – dimethyl biphenyl hybrid was conducted. There are 7 structures dimethyl biphenyl hybrid with only one amino acid include JITYET (Linden & Rippert, 2018a), JITZEU (Linden & Rippert, 2018b), JITYOD (Linden & Rippert, 2018c), NOSPUG & NOSQAN (Weigand et al., 1998), PITSUJ (Linden et al., 2018d) and NIKJOI (Samadi et al., 2013). Seven structures present des torsions angles between dimethyl biphenyl rings from 81.98 to 95.79^o^. They are larger than the angle of title compound.

1. Synthesis and crystallization

To a round-bottom flash was added 6,6'-dimethyl-[1,1'-biphenyl]-2,2'-dicarboxylic acid (1 eq.) and SOCl_2_ (3 eq.) respectively under nitrogen atmosphere. The mixture was heated under reflux for 4 hours and was then evaporated under vacuum. The chloride acid was used for next step without further purification.

To a round-bottom flask was added amine HN–proline– phenylalanine–alanine–COOMe (1 eq.), Et_3_N (2 eq.) and anhydrous CH_2_Cl_2_ (50mL). To this solution was added a solution of (6,6'-dimethyl-[1,1'-biphenyl]-2,2'-dicarbonyl dichloride in CH_2_Cl_2_ at 273 K under an N_2_ atmosphere. After completion of the reaction, the mixture was washed with 1N HCl solution, water and a solution of brine, respectively. The organic phase was dried over Na_2_SO_4_, filtered and evaporated under reduced pressure. The crude product was then purified by flash chromatography (AcOEt/hexane 3:2) to give a white solid (60% yield). The compound was recrystallized by slow evaporation in methanol to give crystals suitable for X-ray diffraction.

1. Refinement

Crystal data, data collection and structure refinement details are summarized in Table 3. Methyl and carboxylic group of Alanine amino acid residue show two conformations with refined occupancy factors converging to 0.502(6) and 0.498(6). Restraints on the geometry and rigid body were applied to the disordered atoms. H atoms were placed at calculated positions (C—H = 0.95–1.08 A˚ and N—H = 0.88 A˚ ), with isotropic displacement parameters Uiso(H) = 1.5Ueq(C) for methyl H atoms and 1.2Ueq(C,N) for all other H atoms. The solvent water molecule is disordered and was refined with a site occupation factor fixed to 0.25. The H atoms of water molecule were located in difference Fourier maps and refined in riding-model approximation with *U*_iso_(H) = 1.5*U*_eq_(O).

Funding information

TTTB thankful to the Asia Research Center-Vietnam National University (ARC-VNU) & Korea Foundation for Advanced Studies (KFAS) for financial support (Project CA.20.7A).

Amine, A., Atmani, Z., Hallaoui, A. E., Giorgi, M., Pierrot, M. & Réglier, M. (2002). *Bioorganic Med. Chem. Lett*. 12(1), 57–60.

Banting, F.G., Best, C. H., Collip, J. B., Campbell, W. R. & Fletcher, A. A. (**1922).** *Can Med Assoc J.* 12 (3), 141–146.

Bruker (2013). APEX2, SAINT and SADABS. Bruker AXS Inc., Madison, Wincousin, USA.

Fosgerau K. & Hoffmann, T. (2015). *Drug Discov Today*. 20, 122–128.

Groom, C. R., Bruno, I. J., Lightfoot, M. P. & Ward, S. C. (2016). Acta Cryst. B72, 171-179.

Hajduk, P. J., Bures, M., Praestgaard, J. & Fesik, S. W. (**2000).** *J. Med. Chem*., 43 (18), 3443 - 3447.

Kaiser, M., Groll, M., Siciliano, C., Machleidt, I. A., Weyher, E., Kohno, J., Milbradt, A. G., Renner, C., Huber, R. & Moroder, L. (2004). ChemBioChem. 5(9), 1256-1266.

Le, T. Q., Nguyen X. T., Nguyen, H. H., Mac, D. H. & Bui, T. T. T. (2020). Acta Cryst E76, 257-260.

Linden, A.& Rippert, A. J. (2018a). Private communication (CCDC reference 1884542). CCDC, Cambridge, England.

Linden, A.& Rippert, A. J. (2018b). Private communication (CCDC reference 1884572). CCDC, Cambridge, England.

Linden, A.& Rippert, A. J. (2018c). Private communication (CCDC reference 1884549). CCDC, Cambridge, England.

Linden, A., Furegati, M.& Rippert, A. J. (2018d). Private communication (CCDC reference 1885480). CCDC, Cambridge, England.

Mann, E., Montero, A., Maestro, M. & Herradón, B. (2002). Helv. Chim. Acta, 85, 3624-3638.

Montero, A., Mann, E., Chana, A. & Herradón, B. (2004a). *Chem. Biodiv*. **2004**, 1, 442-457.

Montero, A., Alberico, F., Royo, M. & Herradón, B. (2004b). Org. Lett. 6, 4089 – 4092.

Otvos, L. Jr. & Wade, J.D. (2014). 2 (62), 1-4.

Ranganathan, D., Kurur, S., Madhusudanan, K. P. & Karle, I. L. (1997). Tetrahedron Lett. 38, 4659-4662.

Samadi, S., Nazari, S., Arvinnezhad, H., Jadidi, K. & Notash, B. (2013). Tetrahedron 69, 6679-6686.

Schimana, J., Gebhardt, K., Holtzel, A., Schmid, D. G., Sussmuth,R., Muller, J., Pukall, R. & Fiedler, H.-P. (2002). J. Antibiot. **55**, 565–570.

Weigand, C., Feigel, M. & Landgrafe, C. (1998). Chem. Commun. 679-680.
