# Supplementary material for: Synthesis and crystal structure of peptide dimethyl biphenyl hybrid C52H60N6O10·0.25H2O
Source: Acta Crystallogr E Crystallogr Commun. 2020 Sep 25;76(Pt 10):1675–8. doi: 10.1107/S2056989020012931 (PMC7534232; doi:10.1107/S2056989020012931)
Supplement: Supplementary file 5 [file e-76-01675-sup6.docx]

**Synthesis and crystal structure of peptide di­methyl bi­phenyl hybrid C_52_H_60_N_6_O_10_·0.25H_2_O**

**Xuan Tu Nguyen, Thuy Quynh Le, Tra My Bui Thi, Dinh Hung Mac and Thai Thanh Thu Bui***

Department of Chemistry, VNU University of science, Vietnam National University, Hanoi, 19 Le Thanh Tong, Hanoi, Vietnam

Correspondence email: thaithanhthubui@gmail.com

**Computing details**

Data collection: Apex 2, Bruker 2013; cell refinement: *SAINT* v8. 34A (Bruker, 2013); data reduction: *SAINT* v8. 34A (Bruker, 2013); program(s) used to solve structure: *SHELXT* (Sheldrick, 2015); program(s) used to refine structure: *SHELXL* (Sheldrick, 2015); molecular graphics: Olex2 (Dolomanov *et al.*, 2009); software used to prepare material for publication: Olex2 (Dolomanov *et al.*, 2009), Mercury (Macrae et *al.,* 2008).

**di­methyl 2,2'-(((2*S*,2'*S*)-2,2'-(((2*S*,2'*S*)-1,1'-(6,6'-di­methyl-[1,1'-bi­phenyl]-2,2'-di­carbonyl)­bis­(pyrrolidine-1,2-diyl-2-carbonyl))bis­(aza­nediyl))bis­(3-phenyl­propanoyl))bis­(aza­nediyl))(2*S*,2'S)-dipropionate**

**Crystal data**

| C_52_H_60_N_6_O_10_·0.25(H_2_O) | *F*(000) = 1986 |
| --- | --- |
| *M_r_* = 933.56 | *D*_x_ = 1.263 Mg m^−3^ |
| Monoclinic, *C*2 | Mo *K*α radiation, λ = 0.71073 Å |
| *a* = 27.505 (3) Å | Cell parameters from 9371 reflections |
| *b* = 12.3814 (12) Å | θ = 2.8–25.8° |
| *c* = 14.6346 (14) Å | µ = 0.09 mm^−1^ |
| β = 99.999 (3)° | *T* = 100 K |
| *V* = 4908.2 (8) Å^3^ | Needle, clear light colourless |
| *Z* = 4 | 0.3 × 0.2 × 0.1 mm |

**Data collection**

| Bruker D8 Quest CMOS diffractometer | 7959 reflections with *I* > 2σ(*I*) |
| --- | --- |
| φ and ω scans | *R*_int_ = 0.062 |
| Absorption correction: multi-scan *SADABS2014*/2 (Bruker,2014/2) was used for absorption correction. wR2(int) was 0.1030 before and 0.0648 after correction. The Ratio of minimum to maximum transmission is 0.9567. The λ/2 correction factor is 0.00150. | θ_max_ = 25.8°, θ_min_ = 2.8° |
| *T*_min_ = 0.713, *T*_max_ = 0.745 | *h* = −33→33 |
| 84318 measured reflections | *k* = −15→15 |
| 9371 independent reflections | *l* = −17→17 |

**Refinement**

| Refinement on *F*^2^ | Hydrogen site location: mixed |
| --- | --- |
| Least-squares matrix: full | H atoms treated by a mixture of independent and constrained refinement |
| *R*[*F*^2^ > 2σ(*F*^2^)] = 0.038 | *w* = 1/[σ^2^(*F*_o_^2^) + (0.0359*P*)^2^ + 2.1291*P*] where *P* = (*F*_o_^2^ + 2*F*_c_^2^)/3 |
| *wR*(*F*^2^) = 0.086 | (Δ/σ)_max_ < 0.001 |
| *S* = 1.06 | Δρ_max_ = 0.28 e Å^−3^ |
| 9371 reflections | Δρ_min_ = −0.16 e Å^−3^ |
| 693 parameters | Absolute structure: Flack x determined using 3323 quotients [(I+)-(I-)]/[(I+)+(I-)] (Parsons, Flack and Wagner, Acta Cryst. B69 (2013) 249-259). |
| 4 restraints | Absolute structure parameter: −0.1 (3) |
| Primary atom site location: dual |  |

**Special details**

| *Geometry*. All e.s.d.'s (except the e.s.d. in the dihedral angle between two l.s. planes) are estimated using the full covariance matrix. The cell e.s.d.'s are taken into account individually in the estimation of e.s.d.'s in distances, angles and torsion angles; correlations between e.s.d.'s in cell parameters are only used when they are defined by crystal symmetry. An approximate (isotropic) treatment of cell e.s.d.'s is used for estimating e.s.d.'s involving l.s. planes. |
| --- |

**Fractional atomic coordinates and isotropic or equivalent isotropic displacement parameters (Å^2^)**

|  | *x* | *y* | *z* | *U*_iso_*/*U*_eq_ | Occ. (<1) |
| --- | --- | --- | --- | --- | --- |
| O5 | 0.33318 (6) | 0.37671 (15) | 0.30911 (12) | 0.0262 (4) |  |
| O4 | 0.25033 (7) | 0.41245 (17) | 0.49754 (15) | 0.0367 (5) |  |
| O6 | 0.37283 (7) | 0.35432 (16) | 0.07943 (13) | 0.0314 (4) |  |
| N3 | 0.36969 (8) | 0.32535 (18) | 0.45153 (14) | 0.0252 (5) |  |
| N2 | 0.32176 (8) | 0.50351 (19) | 0.50273 (15) | 0.0291 (5) |  |
| H2 | 0.3537 | 0.4960 | 0.5038 | 0.035* |  |
| O7 | 0.25946 (8) | 0.5451 (2) | −0.00116 (15) | 0.0531 (6) |  |
| O3 | 0.38550 (8) | 0.64671 (17) | 0.4803 (2) | 0.0552 (7) |  |
| O9 | 0.40799 (7) | 0.7684 (2) | 0.02936 (16) | 0.0536 (7) |  |
| N1 | 0.33544 (8) | 0.7906 (2) | 0.48045 (17) | 0.0351 (6) |  |
| H1 | 0.3070 | 0.8150 | 0.4917 | 0.042* |  |
| N5 | 0.29721 (8) | 0.50901 (18) | 0.14594 (15) | 0.0277 (5) |  |
| H5 | 0.3085 | 0.4583 | 0.1862 | 0.033* |  |
| N4 | 0.29975 (8) | 0.2897 (2) | 0.10755 (15) | 0.0302 (5) |  |
| O2A | 0.4152 (8) | 0.8404 (16) | 0.5865 (9) | 0.052 (4) | 0.498 (6) |
| O10 | 0.47865 (9) | 0.8022 (2) | 0.12726 (19) | 0.0665 (8) |  |
| C19 | 0.37162 (9) | 0.35712 (19) | 0.36445 (17) | 0.0221 (5) |  |
| O8 | 0.36763 (10) | 0.74704 (19) | 0.2248 (2) | 0.0667 (8) |  |
| N6 | 0.38529 (8) | 0.6049 (2) | 0.14123 (16) | 0.0335 (5) |  |
| H6 | 0.3742 | 0.5463 | 0.1106 | 0.040* |  |
| C20 | 0.42128 (9) | 0.3761 (2) | 0.33809 (17) | 0.0224 (5) |  |
| C14 | 0.29481 (10) | 0.4139 (2) | 0.49355 (18) | 0.0277 (6) |  |
| C22 | 0.49273 (10) | 0.4901 (2) | 0.34994 (19) | 0.0300 (6) |  |
| H22 | 0.5116 | 0.5499 | 0.3769 | 0.036* |  |
| C21 | 0.44813 (10) | 0.4663 (2) | 0.37539 (19) | 0.0264 (6) |  |
| H21 | 0.4355 | 0.5113 | 0.4184 | 0.032* |  |
| C39 | 0.27589 (10) | 0.4803 (3) | 0.05996 (19) | 0.0336 (7) |  |
| C34 | 0.34964 (10) | 0.2867 (2) | 0.11713 (18) | 0.0292 (6) |  |
| C25 | 0.43896 (10) | 0.3099 (2) | 0.27349 (17) | 0.0264 (6) |  |
| C15 | 0.32173 (10) | 0.3090 (2) | 0.48016 (18) | 0.0290 (6) |  |
| H15 | 0.3004 | 0.2638 | 0.4327 | 0.035* |  |
| C23 | 0.50992 (10) | 0.4261 (2) | 0.2846 (2) | 0.0339 (7) |  |
| H23 | 0.5403 | 0.4441 | 0.2659 | 0.041* |  |
| C32 | 0.37479 (11) | 0.1926 (2) | 0.1698 (2) | 0.0342 (7) |  |
| C5 | 0.34545 (10) | 0.6858 (2) | 0.4894 (2) | 0.0362 (7) |  |
| C42 | 0.22491 (12) | 0.6148 (3) | 0.2431 (2) | 0.0393 (7) |  |
| C8 | 0.32288 (11) | 0.5941 (3) | 0.6886 (2) | 0.0368 (7) |  |
| C18 | 0.41107 (10) | 0.2861 (3) | 0.52286 (19) | 0.0355 (7) |  |
| H18A | 0.4391 | 0.3375 | 0.5316 | 0.043* |  |
| H18B | 0.4228 | 0.2143 | 0.5062 | 0.043* |  |
| C24 | 0.48386 (10) | 0.3365 (2) | 0.24582 (19) | 0.0326 (7) |  |
| C6 | 0.30370 (9) | 0.6123 (2) | 0.5111 (2) | 0.0303 (6) |  |
| H6A | 0.2743 | 0.6231 | 0.4612 | 0.036* |  |
| C28 | 0.43711 (12) | 0.1101 (2) | 0.2858 (2) | 0.0382 (7) |  |
| C38 | 0.27062 (10) | 0.3606 (3) | 0.03950 (19) | 0.0348 (7) |  |
| H38 | 0.2789 | 0.3459 | −0.0232 | 0.042* |  |
| C27 | 0.41520 (10) | 0.2032 (2) | 0.2421 (2) | 0.0306 (6) |  |
| C7 | 0.28776 (10) | 0.6356 (3) | 0.6048 (2) | 0.0360 (7) |  |
| H7A | 0.2831 (11) | 0.715 (3) | 0.610 (2) | 0.043* |  |
| H7B | 0.2561 (12) | 0.604 (3) | 0.606 (2) | 0.043* |  |
| C40 | 0.30215 (11) | 0.6212 (2) | 0.1744 (2) | 0.0344 (7) |  |
| H40 | 0.2820 | 0.6644 | 0.1237 | 0.041* |  |
| C48 | 0.35480 (12) | 0.6626 (2) | 0.1841 (2) | 0.0395 (7) |  |
| C51 | 0.44291 (12) | 0.7469 (3) | 0.1020 (2) | 0.0465 (8) |  |
| C41 | 0.27949 (12) | 0.6391 (2) | 0.2612 (2) | 0.0403 (7) |  |
| H41A | 0.2962 | 0.5918 | 0.3116 | 0.048* |  |
| H41B | 0.2848 | 0.7150 | 0.2818 | 0.048* |  |
| C49 | 0.43632 (11) | 0.6369 (3) | 0.1441 (2) | 0.0381 (7) |  |
| H49 | 0.4534 | 0.6375 | 0.2103 | 0.046* |  |
| C31 | 0.35836 (13) | 0.0900 (2) | 0.1390 (2) | 0.0456 (8) |  |
| H31 | 0.3315 | 0.0830 | 0.0888 | 0.055* |  |
| C17 | 0.38721 (11) | 0.2796 (3) | 0.6093 (2) | 0.0461 (8) |  |
| H17A | 0.3879 | 0.3506 | 0.6406 | 0.055* |  |
| H17B | 0.4040 | 0.2256 | 0.6538 | 0.055* |  |
| C43 | 0.20546 (13) | 0.5304 (3) | 0.2867 (2) | 0.0406 (8) |  |
| H43 | 0.2267 | 0.4876 | 0.3304 | 0.049* |  |
| C26 | 0.50273 (12) | 0.2713 (3) | 0.1714 (2) | 0.0459 (8) |  |
| H26A | 0.5078 | 0.1962 | 0.1920 | 0.069* |  |
| H26B | 0.5341 | 0.3018 | 0.1604 | 0.069* |  |
| H26C | 0.4785 | 0.2737 | 0.1139 | 0.069* |  |
| C35 | 0.26820 (12) | 0.2261 (3) | 0.1605 (2) | 0.0403 (8) |  |
| H35A | 0.2818 | 0.2267 | 0.2278 | 0.048* |  |
| H35B | 0.2648 | 0.1504 | 0.1386 | 0.048* |  |
| C9 | 0.36689 (11) | 0.6476 (3) | 0.7211 (3) | 0.0515 (9) |  |
| H9 | 0.3753 | 0.7096 | 0.6890 | 0.062* |  |
| C30 | 0.38083 (14) | −0.0007 (3) | 0.1809 (3) | 0.0563 (10) |  |
| H30 | 0.3697 | −0.0703 | 0.1592 | 0.068* |  |
| C36 | 0.21906 (12) | 0.2852 (3) | 0.1399 (2) | 0.0492 (9) |  |
| H36A | 0.2182 | 0.3463 | 0.1833 | 0.059* |  |
| H36B | 0.1912 | 0.2358 | 0.1441 | 0.059* |  |
| C44 | 0.15538 (13) | 0.5070 (3) | 0.2676 (2) | 0.0488 (9) |  |
| H44 | 0.1427 | 0.4490 | 0.2990 | 0.059* |  |
| C16 | 0.33427 (11) | 0.2449 (3) | 0.5705 (2) | 0.0434 (8) |  |
| H16A | 0.3324 | 0.1662 | 0.5584 | 0.052* |  |
| H16B | 0.3117 | 0.2637 | 0.6138 | 0.052* |  |
| C29 | 0.41922 (14) | 0.0090 (3) | 0.2539 (2) | 0.0520 (9) |  |
| H29 | 0.4339 | −0.0543 | 0.2834 | 0.062* |  |
| C33 | 0.48036 (13) | 0.1160 (3) | 0.3656 (2) | 0.0466 (8) |  |
| H33A | 0.5112 | 0.1201 | 0.3410 | 0.070* |  |
| H33B | 0.4807 | 0.0514 | 0.4044 | 0.070* |  |
| H33C | 0.4771 | 0.1804 | 0.4030 | 0.070* |  |
| C37 | 0.21720 (11) | 0.3253 (3) | 0.0408 (2) | 0.0492 (9) |  |
| H37A | 0.1941 | 0.3867 | 0.0268 | 0.059* |  |
| H37B | 0.2070 | 0.2668 | −0.0047 | 0.059* |  |
| C50 | 0.46145 (12) | 0.5544 (3) | 0.0915 (2) | 0.0498 (9) |  |
| H50A | 0.4560 | 0.4818 | 0.1145 | 0.075* |  |
| H50B | 0.4970 | 0.5693 | 0.1008 | 0.075* |  |
| H50C | 0.4476 | 0.5587 | 0.0252 | 0.075* |  |
| C13 | 0.31123 (13) | 0.5060 (3) | 0.7362 (2) | 0.0546 (9) |  |
| H13 | 0.2811 | 0.4689 | 0.7158 | 0.065* |  |
| C3 | 0.37093 (12) | 0.8660 (3) | 0.4522 (3) | 0.0506 (9) |  |
| H3A | 0.3893 | 0.8282 | 0.4082 | 0.061* | 0.498 (6) |
| H3B | 0.3776 | 0.8469 | 0.3891 | 0.061* | 0.502 (6) |
| C45 | 0.12403 (14) | 0.5668 (3) | 0.2040 (3) | 0.0587 (10) |  |
| H45 | 0.0898 | 0.5502 | 0.1903 | 0.070* |  |
| C47 | 0.19287 (15) | 0.6750 (4) | 0.1803 (3) | 0.0668 (12) |  |
| H47 | 0.2053 | 0.7342 | 0.1500 | 0.080* |  |
| C52 | 0.41693 (14) | 0.8637 (4) | −0.0232 (3) | 0.0722 (13) |  |
| H52A | 0.3905 | 0.8713 | −0.0770 | 0.108* |  |
| H52B | 0.4487 | 0.8563 | −0.0443 | 0.108* |  |
| H52C | 0.4177 | 0.9278 | 0.0163 | 0.108* |  |
| C11 | 0.38662 (15) | 0.5233 (4) | 0.8454 (3) | 0.0675 (12) |  |
| H11 | 0.4085 | 0.4988 | 0.8989 | 0.081* |  |
| C10 | 0.39836 (14) | 0.6128 (4) | 0.7984 (3) | 0.0665 (12) |  |
| H10 | 0.4283 | 0.6505 | 0.8195 | 0.080* |  |
| C12 | 0.34359 (16) | 0.4694 (4) | 0.8155 (3) | 0.0695 (12) |  |
| H12 | 0.3354 | 0.4074 | 0.8480 | 0.083* |  |
| C46 | 0.14300 (16) | 0.6512 (4) | 0.1605 (3) | 0.0794 (14) |  |
| H46 | 0.1217 | 0.6935 | 0.1164 | 0.095* |  |
| O1B | 0.46376 (18) | 0.8694 (5) | 0.5125 (5) | 0.082 (2) | 0.502 (6) |
| C1A | 0.4577 (7) | 0.8657 (13) | 0.6565 (10) | 0.065 (4) | 0.498 (6) |
| H1AA | 0.4865 | 0.8781 | 0.6266 | 0.098* | 0.498 (6) |
| H1AB | 0.4645 | 0.8052 | 0.7000 | 0.098* | 0.498 (6) |
| H1AC | 0.4510 | 0.9308 | 0.6902 | 0.098* | 0.498 (6) |
| C4A | 0.3372 (3) | 0.9666 (6) | 0.3947 (6) | 0.0338 (16) | 0.498 (6) |
| H4AA | 0.314 (3) | 1.001 (6) | 0.437 (5) | 0.051* | 0.498 (6) |
| H4AB | 0.318 (3) | 0.951 (6) | 0.348 (5) | 0.051* | 0.498 (6) |
| H4AC | 0.365 (3) | 1.022 (6) | 0.380 (5) | 0.051* | 0.498 (6) |
| C2B | 0.4233 (4) | 0.8525 (9) | 0.5327 (8) | 0.051 (3) | 0.502 (6) |
| O11 | 0.2727 (2) | 0.8908 (5) | 0.1778 (4) | 0.0166 (14) | 0.25 |
| H11A | 0.3048 | 0.8916 | 0.1906 | 0.025* | 0.25 |
| H11B | 0.2657 | 0.8381 | 0.1383 | 0.025* | 0.25 |
| C4B | 0.3574 (3) | 0.9738 (5) | 0.4551 (7) | 0.049 (2) | 0.502 (6) |
| H4BA | 0.3299 | 0.9887 | 0.4044 | 0.074* | 0.502 (6) |
| H4BB | 0.3856 | 1.0197 | 0.4483 | 0.074* | 0.502 (6) |
| H4BC | 0.3471 | 0.9891 | 0.5146 | 0.074* | 0.502 (6) |
| C2A | 0.4049 (2) | 0.9141 (6) | 0.5214 (4) | 0.0317 (15) | 0.498 (6) |
| O2B | 0.4184 (6) | 0.8407 (16) | 0.6190 (9) | 0.044 (3) | 0.502 (6) |
| O1A | 0.42431 (16) | 1.0015 (3) | 0.5223 (3) | 0.0492 (16) | 0.498 (6) |
| C1B | 0.4595 (6) | 0.8377 (14) | 0.6925 (11) | 0.081 (5) | 0.502 (6) |
| H1BA | 0.4832 | 0.7835 | 0.6788 | 0.121* | 0.502 (6) |
| H1BB | 0.4483 | 0.8188 | 0.7504 | 0.121* | 0.502 (6) |
| H1BC | 0.4754 | 0.9088 | 0.6989 | 0.121* | 0.502 (6) |

**Atomic displacement parameters (Å^2^)**

|  | *U*^11^ | *U*^22^ | *U*^33^ | *U*^12^ | *U*^13^ | *U*^23^ |
| --- | --- | --- | --- | --- | --- | --- |
| O5 | 0.0269 (9) | 0.0271 (10) | 0.0227 (9) | 0.0019 (8) | −0.0009 (8) | −0.0012 (8) |
| O4 | 0.0254 (10) | 0.0376 (12) | 0.0472 (12) | −0.0104 (9) | 0.0069 (8) | −0.0008 (10) |
| O6 | 0.0302 (10) | 0.0322 (11) | 0.0318 (10) | 0.0050 (9) | 0.0054 (8) | −0.0008 (9) |
| N3 | 0.0254 (12) | 0.0256 (12) | 0.0232 (11) | −0.0044 (9) | 0.0002 (9) | 0.0021 (9) |
| N2 | 0.0221 (11) | 0.0308 (13) | 0.0348 (13) | −0.0061 (10) | 0.0056 (9) | −0.0074 (11) |
| O7 | 0.0503 (14) | 0.0743 (17) | 0.0343 (11) | 0.0200 (12) | 0.0064 (10) | 0.0256 (12) |
| O3 | 0.0292 (12) | 0.0309 (12) | 0.114 (2) | −0.0076 (9) | 0.0349 (13) | −0.0243 (13) |
| O9 | 0.0327 (12) | 0.0701 (18) | 0.0595 (15) | 0.0071 (11) | 0.0126 (11) | 0.0410 (13) |
| N1 | 0.0259 (12) | 0.0284 (13) | 0.0554 (16) | −0.0076 (10) | 0.0196 (11) | −0.0116 (12) |
| N5 | 0.0354 (13) | 0.0248 (12) | 0.0246 (12) | 0.0065 (10) | 0.0099 (10) | 0.0053 (10) |
| N4 | 0.0309 (12) | 0.0303 (13) | 0.0290 (12) | −0.0028 (10) | 0.0040 (10) | −0.0103 (10) |
| O2A | 0.041 (5) | 0.038 (5) | 0.065 (10) | −0.016 (3) | −0.026 (7) | 0.003 (8) |
| O10 | 0.0492 (15) | 0.0653 (18) | 0.0834 (19) | −0.0116 (14) | 0.0069 (13) | 0.0320 (15) |
| C19 | 0.0295 (14) | 0.0124 (12) | 0.0231 (13) | −0.0013 (10) | 0.0014 (11) | −0.0028 (10) |
| O8 | 0.0794 (18) | 0.0229 (12) | 0.110 (2) | −0.0139 (12) | 0.0500 (16) | −0.0127 (13) |
| N6 | 0.0345 (13) | 0.0342 (13) | 0.0325 (13) | −0.0013 (11) | 0.0081 (10) | 0.0046 (11) |
| C20 | 0.0277 (13) | 0.0161 (12) | 0.0222 (13) | 0.0028 (10) | 0.0005 (10) | 0.0058 (11) |
| C14 | 0.0282 (15) | 0.0322 (15) | 0.0219 (14) | −0.0075 (12) | 0.0020 (11) | 0.0003 (12) |
| C22 | 0.0305 (15) | 0.0233 (15) | 0.0342 (15) | −0.0039 (12) | 0.0000 (12) | 0.0053 (12) |
| C21 | 0.0305 (15) | 0.0200 (13) | 0.0269 (14) | 0.0013 (11) | 0.0000 (11) | 0.0012 (11) |
| C39 | 0.0246 (14) | 0.052 (2) | 0.0257 (15) | 0.0092 (13) | 0.0095 (12) | 0.0095 (14) |
| C34 | 0.0337 (15) | 0.0254 (14) | 0.0272 (14) | 0.0013 (12) | 0.0019 (12) | −0.0103 (12) |
| C25 | 0.0307 (14) | 0.0229 (14) | 0.0241 (13) | 0.0047 (11) | 0.0006 (11) | 0.0036 (11) |
| C15 | 0.0274 (14) | 0.0286 (15) | 0.0300 (15) | −0.0091 (11) | 0.0020 (11) | 0.0045 (12) |
| C23 | 0.0269 (14) | 0.0369 (17) | 0.0379 (17) | −0.0005 (13) | 0.0057 (12) | 0.0097 (14) |
| C32 | 0.0427 (17) | 0.0248 (15) | 0.0337 (16) | 0.0039 (13) | 0.0028 (13) | −0.0065 (12) |
| C5 | 0.0258 (15) | 0.0318 (17) | 0.054 (2) | −0.0077 (12) | 0.0160 (14) | −0.0184 (14) |
| C42 | 0.0492 (18) | 0.0316 (16) | 0.0422 (17) | 0.0097 (14) | 0.0227 (14) | −0.0042 (14) |
| C8 | 0.0328 (15) | 0.0426 (18) | 0.0371 (16) | 0.0007 (13) | 0.0116 (13) | −0.0186 (15) |
| C18 | 0.0313 (15) | 0.0407 (17) | 0.0315 (15) | −0.0012 (13) | −0.0032 (12) | 0.0135 (14) |
| C24 | 0.0320 (15) | 0.0343 (17) | 0.0319 (15) | 0.0077 (13) | 0.0070 (12) | 0.0033 (13) |
| C6 | 0.0210 (13) | 0.0302 (15) | 0.0406 (16) | −0.0080 (12) | 0.0084 (11) | −0.0110 (13) |
| C28 | 0.0498 (18) | 0.0244 (15) | 0.0389 (17) | 0.0049 (14) | 0.0033 (14) | −0.0024 (14) |
| C38 | 0.0255 (14) | 0.057 (2) | 0.0208 (13) | 0.0045 (14) | 0.0019 (11) | −0.0098 (14) |
| C27 | 0.0378 (16) | 0.0226 (14) | 0.0316 (15) | 0.0033 (12) | 0.0069 (12) | −0.0036 (12) |
| C7 | 0.0213 (14) | 0.043 (2) | 0.0457 (18) | −0.0077 (13) | 0.0109 (13) | −0.0152 (15) |
| C40 | 0.0463 (17) | 0.0194 (15) | 0.0407 (16) | 0.0077 (12) | 0.0168 (13) | 0.0088 (12) |
| C48 | 0.055 (2) | 0.0228 (16) | 0.0453 (18) | 0.0042 (14) | 0.0211 (15) | 0.0123 (14) |
| C51 | 0.0326 (17) | 0.057 (2) | 0.051 (2) | 0.0011 (16) | 0.0109 (15) | 0.0229 (17) |
| C41 | 0.058 (2) | 0.0239 (16) | 0.0442 (18) | 0.0049 (14) | 0.0228 (15) | 0.0003 (13) |
| C49 | 0.0352 (16) | 0.0478 (19) | 0.0303 (15) | −0.0007 (14) | 0.0030 (12) | 0.0116 (14) |
| C31 | 0.059 (2) | 0.0280 (18) | 0.0443 (19) | 0.0017 (15) | −0.0071 (15) | −0.0127 (14) |
| C17 | 0.0407 (17) | 0.065 (2) | 0.0304 (16) | −0.0062 (16) | −0.0007 (13) | 0.0203 (16) |
| C43 | 0.056 (2) | 0.0351 (18) | 0.0337 (16) | 0.0022 (15) | 0.0160 (14) | −0.0066 (14) |
| C26 | 0.0419 (18) | 0.052 (2) | 0.0469 (19) | 0.0102 (16) | 0.0174 (15) | −0.0010 (16) |
| C35 | 0.0485 (19) | 0.0351 (17) | 0.0394 (17) | −0.0185 (15) | 0.0138 (14) | −0.0148 (14) |
| C9 | 0.0289 (17) | 0.046 (2) | 0.075 (2) | 0.0047 (14) | −0.0034 (16) | −0.0197 (18) |
| C30 | 0.079 (3) | 0.0222 (17) | 0.060 (2) | 0.0023 (17) | −0.0101 (19) | −0.0131 (16) |
| C36 | 0.0418 (18) | 0.049 (2) | 0.062 (2) | −0.0186 (16) | 0.0242 (16) | −0.0270 (18) |
| C44 | 0.060 (2) | 0.042 (2) | 0.051 (2) | −0.0074 (17) | 0.0283 (17) | −0.0189 (17) |
| C16 | 0.0408 (18) | 0.051 (2) | 0.0391 (18) | −0.0072 (15) | 0.0079 (14) | 0.0166 (15) |
| C29 | 0.071 (2) | 0.0218 (16) | 0.058 (2) | 0.0099 (16) | −0.0032 (18) | −0.0010 (16) |
| C33 | 0.057 (2) | 0.0244 (16) | 0.052 (2) | 0.0060 (15) | −0.0065 (16) | 0.0037 (15) |
| C37 | 0.0285 (16) | 0.066 (2) | 0.052 (2) | −0.0036 (15) | 0.0053 (14) | −0.0270 (18) |
| C50 | 0.0316 (17) | 0.073 (2) | 0.0423 (19) | 0.0083 (17) | 0.0006 (14) | 0.0043 (18) |
| C13 | 0.055 (2) | 0.075 (3) | 0.0339 (18) | −0.022 (2) | 0.0101 (15) | −0.0034 (18) |
| C3 | 0.048 (2) | 0.0403 (19) | 0.073 (2) | −0.0194 (16) | 0.0389 (18) | −0.0190 (18) |
| C45 | 0.047 (2) | 0.069 (3) | 0.063 (2) | 0.009 (2) | 0.0205 (19) | −0.013 (2) |
| C47 | 0.060 (2) | 0.064 (3) | 0.085 (3) | 0.023 (2) | 0.035 (2) | 0.034 (2) |
| C52 | 0.047 (2) | 0.088 (3) | 0.089 (3) | 0.017 (2) | 0.032 (2) | 0.066 (3) |
| C11 | 0.059 (3) | 0.093 (4) | 0.046 (2) | 0.008 (2) | −0.0040 (18) | −0.026 (2) |
| C10 | 0.042 (2) | 0.062 (3) | 0.087 (3) | 0.0087 (19) | −0.013 (2) | −0.031 (2) |
| C12 | 0.079 (3) | 0.091 (3) | 0.039 (2) | −0.013 (2) | 0.013 (2) | 0.005 (2) |
| C46 | 0.053 (3) | 0.095 (4) | 0.094 (3) | 0.031 (2) | 0.022 (2) | 0.029 (3) |
| O1B | 0.034 (3) | 0.090 (5) | 0.129 (6) | −0.012 (3) | 0.029 (3) | 0.011 (4) |
| C1A | 0.067 (8) | 0.054 (7) | 0.063 (8) | −0.011 (6) | −0.020 (6) | 0.018 (5) |
| C4A | 0.029 (4) | 0.035 (4) | 0.038 (4) | 0.007 (3) | 0.007 (3) | 0.009 (3) |
| C2B | 0.040 (5) | 0.023 (5) | 0.095 (9) | −0.004 (4) | 0.027 (6) | −0.008 (5) |
| O11 | 0.016 (3) | 0.015 (3) | 0.019 (3) | −0.002 (3) | 0.005 (3) | −0.001 (3) |
| C4B | 0.051 (5) | 0.035 (4) | 0.069 (6) | −0.002 (3) | 0.028 (5) | 0.012 (4) |
| C2A | 0.024 (3) | 0.025 (4) | 0.048 (4) | 0.000 (3) | 0.012 (3) | 0.001 (3) |
| O2B | 0.023 (4) | 0.034 (4) | 0.065 (8) | −0.008 (3) | −0.017 (6) | −0.012 (6) |
| O1A | 0.041 (3) | 0.027 (3) | 0.074 (3) | −0.017 (2) | −0.006 (2) | 0.009 (2) |
| C1B | 0.039 (5) | 0.087 (11) | 0.101 (12) | −0.018 (6) | −0.031 (7) | −0.012 (9) |

**Geometric parameters (Å, º)**

| O5—C19 | 1.239 (3) | C49—C50 | 1.517 (5) |
| --- | --- | --- | --- |
| O4—C14 | 1.235 (3) | C31—H31 | 0.9500 |
| O6—C34 | 1.239 (3) | C31—C30 | 1.374 (5) |
| N3—C19 | 1.343 (3) | C17—H17A | 0.9900 |
| N3—C15 | 1.466 (3) | C17—H17B | 0.9900 |
| N3—C18 | 1.486 (3) | C17—C16 | 1.530 (4) |
| N2—H2 | 0.8800 | C43—H43 | 0.9500 |
| N2—C14 | 1.328 (3) | C43—C44 | 1.388 (5) |
| N2—C6 | 1.449 (4) | C26—H26A | 0.9800 |
| O7—C39 | 1.228 (4) | C26—H26B | 0.9800 |
| O3—C5 | 1.232 (3) | C26—H26C | 0.9800 |
| O9—C51 | 1.330 (4) | C35—H35A | 0.9900 |
| O9—C52 | 1.452 (4) | C35—H35B | 0.9900 |
| N1—H1 | 0.8800 | C35—C36 | 1.521 (5) |
| N1—C5 | 1.329 (4) | C9—H9 | 0.9500 |
| N1—C3 | 1.461 (4) | C9—C10 | 1.369 (5) |
| N5—H5 | 0.8800 | C30—H30 | 0.9500 |
| N5—C39 | 1.341 (4) | C30—C29 | 1.371 (5) |
| N5—C40 | 1.450 (4) | C36—H36A | 0.9900 |
| N4—C34 | 1.355 (3) | C36—H36B | 0.9900 |
| N4—C38 | 1.459 (4) | C36—C37 | 1.526 (5) |
| N4—C35 | 1.486 (4) | C44—H44 | 0.9500 |
| O2A—C1A | 1.449 (14) | C44—C45 | 1.371 (5) |
| O2A—C2A | 1.314 (15) | C16—H16A | 0.9900 |
| O10—C51 | 1.203 (4) | C16—H16B | 0.9900 |
| C19—C20 | 1.501 (4) | C29—H29 | 0.9500 |
| O8—C48 | 1.225 (4) | C33—H33A | 0.9800 |
| N6—H6 | 0.8800 | C33—H33B | 0.9800 |
| N6—C48 | 1.338 (4) | C33—H33C | 0.9800 |
| N6—C49 | 1.452 (4) | C37—H37A | 0.9900 |
| C20—C21 | 1.397 (4) | C37—H37B | 0.9900 |
| C20—C25 | 1.401 (4) | C50—H50A | 0.9800 |
| C14—C15 | 1.524 (4) | C50—H50B | 0.9800 |
| C22—H22 | 0.9500 | C50—H50C | 0.9800 |
| C22—C21 | 1.375 (4) | C13—H13 | 0.9500 |
| C22—C23 | 1.387 (4) | C13—C12 | 1.409 (5) |
| C21—H21 | 0.9500 | C3—H3A | 1.0000 |
| C39—C38 | 1.513 (5) | C3—H3B | 1.0000 |
| C34—C32 | 1.498 (4) | C3—C4A | 1.688 (8) |
| C25—C24 | 1.404 (4) | C3—C2B | 1.704 (13) |
| C25—C27 | 1.510 (4) | C3—C4B | 1.389 (7) |
| C15—H15 | 1.0000 | C3—C2A | 1.388 (7) |
| C15—C16 | 1.529 (4) | C45—H45 | 0.9500 |
| C23—H23 | 0.9500 | C45—C46 | 1.373 (6) |
| C23—C24 | 1.388 (4) | C47—H47 | 0.9500 |
| C32—C27 | 1.402 (4) | C47—C46 | 1.384 (6) |
| C32—C31 | 1.396 (4) | C52—H52A | 0.9800 |
| C5—C6 | 1.540 (4) | C52—H52B | 0.9800 |
| C42—C41 | 1.509 (5) | C52—H52C | 0.9800 |
| C42—C43 | 1.379 (4) | C11—H11 | 0.9500 |
| C42—C47 | 1.377 (5) | C11—C10 | 1.372 (6) |
| C8—C7 | 1.513 (4) | C11—C12 | 1.363 (6) |
| C8—C9 | 1.389 (4) | C10—H10 | 0.9500 |
| C8—C13 | 1.363 (5) | C12—H12 | 0.9500 |
| C18—H18A | 0.9900 | C46—H46 | 0.9500 |
| C18—H18B | 0.9900 | O1B—C2B | 1.218 (10) |
| C18—C17 | 1.526 (4) | C1A—H1AA | 0.9800 |
| C24—C26 | 1.517 (4) | C1A—H1AB | 0.9800 |
| C6—H6A | 1.0000 | C1A—H1AC | 0.9800 |
| C6—C7 | 1.537 (4) | C4A—H4AA | 1.05 (7) |
| C28—C27 | 1.403 (4) | C4A—H4AB | 0.81 (8) |
| C28—C29 | 1.395 (5) | C4A—H4AC | 1.08 (8) |
| C28—C33 | 1.517 (4) | C2B—O2B | 1.301 (17) |
| C38—H38 | 1.0000 | O11—H11A | 0.8705 |
| C38—C37 | 1.537 (4) | O11—H11B | 0.8699 |
| C7—H7A | 0.99 (4) | C4B—H4BA | 0.9800 |
| C7—H7B | 0.96 (3) | C4B—H4BB | 0.9800 |
| C40—H40 | 1.0000 | C4B—H4BC | 0.9800 |
| C40—C48 | 1.519 (4) | C2A—O1A | 1.205 (8) |
| C40—C41 | 1.525 (4) | O2B—C1B | 1.419 (13) |
| C51—C49 | 1.518 (5) | C1B—H1BA | 0.9800 |
| C41—H41A | 0.9900 | C1B—H1BB | 0.9800 |
| C41—H41B | 0.9900 | C1B—H1BC | 0.9800 |
| C49—H49 | 1.0000 |  |  |
|  |  |  |  |
| C19—N3—C15 | 119.8 (2) | C16—C17—H17B | 111.2 |
| C19—N3—C18 | 127.6 (2) | C42—C43—H43 | 119.4 |
| C15—N3—C18 | 111.8 (2) | C42—C43—C44 | 121.1 (3) |
| C14—N2—H2 | 116.9 | C44—C43—H43 | 119.4 |
| C14—N2—C6 | 126.3 (2) | C24—C26—H26A | 109.5 |
| C6—N2—H2 | 116.9 | C24—C26—H26B | 109.5 |
| C51—O9—C52 | 114.9 (3) | C24—C26—H26C | 109.5 |
| C5—N1—H1 | 119.5 | H26A—C26—H26B | 109.5 |
| C5—N1—C3 | 121.0 (2) | H26A—C26—H26C | 109.5 |
| C3—N1—H1 | 119.5 | H26B—C26—H26C | 109.5 |
| C39—N5—H5 | 119.1 | N4—C35—H35A | 111.2 |
| C39—N5—C40 | 121.9 (2) | N4—C35—H35B | 111.2 |
| C40—N5—H5 | 119.1 | N4—C35—C36 | 102.8 (3) |
| C34—N4—C38 | 120.9 (2) | H35A—C35—H35B | 109.1 |
| C34—N4—C35 | 127.2 (3) | C36—C35—H35A | 111.2 |
| C38—N4—C35 | 112.0 (2) | C36—C35—H35B | 111.2 |
| C2A—O2A—C1A | 114.1 (14) | C8—C9—H9 | 119.3 |
| O5—C19—N3 | 120.5 (2) | C10—C9—C8 | 121.4 (4) |
| O5—C19—C20 | 120.8 (2) | C10—C9—H9 | 119.3 |
| N3—C19—C20 | 118.5 (2) | C31—C30—H30 | 119.9 |
| C48—N6—H6 | 119.3 | C29—C30—C31 | 120.2 (3) |
| C48—N6—C49 | 121.5 (3) | C29—C30—H30 | 119.9 |
| C49—N6—H6 | 119.3 | C35—C36—H36A | 111.1 |
| C21—C20—C19 | 117.9 (2) | C35—C36—H36B | 111.1 |
| C21—C20—C25 | 120.6 (2) | C35—C36—C37 | 103.1 (2) |
| C25—C20—C19 | 121.3 (2) | H36A—C36—H36B | 109.1 |
| O4—C14—N2 | 123.2 (3) | C37—C36—H36A | 111.1 |
| O4—C14—C15 | 120.1 (2) | C37—C36—H36B | 111.1 |
| N2—C14—C15 | 116.7 (2) | C43—C44—H44 | 119.7 |
| C21—C22—H22 | 120.3 | C45—C44—C43 | 120.6 (3) |
| C21—C22—C23 | 119.4 (3) | C45—C44—H44 | 119.7 |
| C23—C22—H22 | 120.3 | C15—C16—C17 | 103.5 (2) |
| C20—C21—H21 | 119.9 | C15—C16—H16A | 111.1 |
| C22—C21—C20 | 120.1 (3) | C15—C16—H16B | 111.1 |
| C22—C21—H21 | 119.9 | C17—C16—H16A | 111.1 |
| O7—C39—N5 | 123.8 (3) | C17—C16—H16B | 111.1 |
| O7—C39—C38 | 119.0 (3) | H16A—C16—H16B | 109.0 |
| N5—C39—C38 | 117.2 (2) | C28—C29—H29 | 119.4 |
| O6—C34—N4 | 121.7 (3) | C30—C29—C28 | 121.2 (3) |
| O6—C34—C32 | 121.8 (2) | C30—C29—H29 | 119.4 |
| N4—C34—C32 | 116.3 (3) | C28—C33—H33A | 109.5 |
| C20—C25—C24 | 118.9 (2) | C28—C33—H33B | 109.5 |
| C20—C25—C27 | 122.3 (2) | C28—C33—H33C | 109.5 |
| C24—C25—C27 | 118.3 (2) | H33A—C33—H33B | 109.5 |
| N3—C15—C14 | 113.6 (2) | H33A—C33—H33C | 109.5 |
| N3—C15—H15 | 109.1 | H33B—C33—H33C | 109.5 |
| N3—C15—C16 | 103.9 (2) | C38—C37—H37A | 111.1 |
| C14—C15—H15 | 109.1 | C38—C37—H37B | 111.1 |
| C14—C15—C16 | 111.9 (2) | C36—C37—C38 | 103.3 (2) |
| C16—C15—H15 | 109.1 | C36—C37—H37A | 111.1 |
| C22—C23—H23 | 119.2 | C36—C37—H37B | 111.1 |
| C22—C23—C24 | 121.7 (3) | H37A—C37—H37B | 109.1 |
| C24—C23—H23 | 119.2 | C49—C50—H50A | 109.5 |
| C27—C32—C34 | 123.4 (2) | C49—C50—H50B | 109.5 |
| C31—C32—C34 | 116.5 (3) | C49—C50—H50C | 109.5 |
| C31—C32—C27 | 120.0 (3) | H50A—C50—H50B | 109.5 |
| O3—C5—N1 | 123.2 (3) | H50A—C50—H50C | 109.5 |
| O3—C5—C6 | 120.2 (3) | H50B—C50—H50C | 109.5 |
| N1—C5—C6 | 116.6 (2) | C8—C13—H13 | 119.7 |
| C43—C42—C41 | 121.6 (3) | C8—C13—C12 | 120.5 (3) |
| C47—C42—C41 | 120.7 (3) | C12—C13—H13 | 119.7 |
| C47—C42—C43 | 117.7 (3) | N1—C3—H3A | 108.7 |
| C9—C8—C7 | 120.8 (3) | N1—C3—H3B | 110.4 |
| C13—C8—C7 | 120.8 (3) | N1—C3—C4A | 106.0 (3) |
| C13—C8—C9 | 118.3 (3) | N1—C3—C2B | 105.5 (4) |
| N3—C18—H18A | 111.3 | C4A—C3—H3A | 108.7 |
| N3—C18—H18B | 111.3 | C2B—C3—H3B | 110.4 |
| N3—C18—C17 | 102.3 (2) | C4B—C3—N1 | 114.2 (4) |
| H18A—C18—H18B | 109.2 | C4B—C3—H3B | 110.4 |
| C17—C18—H18A | 111.3 | C4B—C3—C2B | 105.8 (6) |
| C17—C18—H18B | 111.3 | C2A—C3—N1 | 117.8 (4) |
| C25—C24—C26 | 120.6 (3) | C2A—C3—H3A | 108.7 |
| C23—C24—C25 | 119.2 (3) | C2A—C3—C4A | 106.8 (5) |
| C23—C24—C26 | 120.2 (3) | C44—C45—H45 | 120.6 |
| N2—C6—C5 | 104.6 (2) | C44—C45—C46 | 118.8 (4) |
| N2—C6—H6A | 107.9 | C46—C45—H45 | 120.6 |
| N2—C6—C7 | 113.9 (3) | C42—C47—H47 | 119.3 |
| C5—C6—H6A | 107.9 | C42—C47—C46 | 121.3 (4) |
| C7—C6—C5 | 114.3 (2) | C46—C47—H47 | 119.3 |
| C7—C6—H6A | 107.9 | O9—C52—H52A | 109.5 |
| C27—C28—C33 | 121.9 (3) | O9—C52—H52B | 109.5 |
| C29—C28—C27 | 119.1 (3) | O9—C52—H52C | 109.5 |
| C29—C28—C33 | 119.0 (3) | H52A—C52—H52B | 109.5 |
| N4—C38—C39 | 115.6 (2) | H52A—C52—H52C | 109.5 |
| N4—C38—H38 | 109.3 | H52B—C52—H52C | 109.5 |
| N4—C38—C37 | 103.5 (3) | C10—C11—H11 | 120.0 |
| C39—C38—H38 | 109.3 | C12—C11—H11 | 120.0 |
| C39—C38—C37 | 109.6 (2) | C12—C11—C10 | 120.1 (4) |
| C37—C38—H38 | 109.3 | C9—C10—C11 | 119.9 (4) |
| C32—C27—C25 | 123.8 (2) | C9—C10—H10 | 120.1 |
| C32—C27—C28 | 119.2 (3) | C11—C10—H10 | 120.1 |
| C28—C27—C25 | 116.9 (2) | C13—C12—H12 | 120.1 |
| C8—C7—C6 | 114.8 (2) | C11—C12—C13 | 119.8 (4) |
| C8—C7—H7A | 110.1 (19) | C11—C12—H12 | 120.1 |
| C8—C7—H7B | 107.6 (19) | C45—C46—C47 | 120.5 (4) |
| C6—C7—H7A | 108.4 (19) | C45—C46—H46 | 119.7 |
| C6—C7—H7B | 109.5 (19) | C47—C46—H46 | 119.7 |
| H7A—C7—H7B | 106 (3) | O2A—C1A—H1AA | 109.5 |
| N5—C40—H40 | 106.6 | O2A—C1A—H1AB | 109.5 |
| N5—C40—C48 | 113.0 (2) | O2A—C1A—H1AC | 109.5 |
| N5—C40—C41 | 110.3 (2) | H1AA—C1A—H1AB | 109.5 |
| C48—C40—H40 | 106.6 | H1AA—C1A—H1AC | 109.5 |
| C48—C40—C41 | 113.2 (3) | H1AB—C1A—H1AC | 109.5 |
| C41—C40—H40 | 106.6 | C3—C4A—H4AA | 110 (4) |
| O8—C48—N6 | 122.2 (3) | C3—C4A—H4AB | 117 (5) |
| O8—C48—C40 | 121.5 (3) | C3—C4A—H4AC | 102 (4) |
| N6—C48—C40 | 116.2 (3) | H4AA—C4A—H4AB | 102 (6) |
| O9—C51—C49 | 112.6 (3) | H4AA—C4A—H4AC | 113 (5) |
| O10—C51—O9 | 124.8 (3) | H4AB—C4A—H4AC | 112 (7) |
| O10—C51—C49 | 122.3 (3) | O1B—C2B—C3 | 120.9 (9) |
| C42—C41—C40 | 111.3 (3) | O1B—C2B—O2B | 120.6 (13) |
| C42—C41—H41A | 109.4 | O2B—C2B—C3 | 117.7 (9) |
| C42—C41—H41B | 109.4 | H11A—O11—H11B | 104.4 |
| C40—C41—H41A | 109.4 | C3—C4B—H4BA | 109.5 |
| C40—C41—H41B | 109.4 | C3—C4B—H4BB | 109.5 |
| H41A—C41—H41B | 108.0 | C3—C4B—H4BC | 109.5 |
| N6—C49—C51 | 114.6 (2) | H4BA—C4B—H4BB | 109.5 |
| N6—C49—H49 | 108.6 | H4BA—C4B—H4BC | 109.5 |
| N6—C49—C50 | 108.9 (3) | H4BB—C4B—H4BC | 109.5 |
| C51—C49—H49 | 108.6 | O2A—C2A—C3 | 105.3 (9) |
| C50—C49—C51 | 107.5 (3) | O1A—C2A—O2A | 125.2 (10) |
| C50—C49—H49 | 108.6 | O1A—C2A—C3 | 129.3 (6) |
| C32—C31—H31 | 119.9 | C2B—O2B—C1B | 122.3 (15) |
| C30—C31—C32 | 120.2 (3) | O2B—C1B—H1BA | 109.5 |
| C30—C31—H31 | 119.9 | O2B—C1B—H1BB | 109.5 |
| C18—C17—H17A | 111.2 | O2B—C1B—H1BC | 109.5 |
| C18—C17—H17B | 111.2 | H1BA—C1B—H1BB | 109.5 |
| C18—C17—C16 | 103.0 (2) | H1BA—C1B—H1BC | 109.5 |
| H17A—C17—H17B | 109.1 | H1BB—C1B—H1BC | 109.5 |
| C16—C17—H17A | 111.2 |  |  |
