# Supplementary figures and images for: Synthesis and crystal structure of peptide dimethyl biphenyl hybrid C52H60N6O10·0.25H2O
Source: Acta Crystallogr E Crystallogr Commun. 2020 Sep 25;76(Pt 10):1675–8. doi: 10.1107/S2056989020012931 (PMC7534232; doi:10.1107/S2056989020012931)

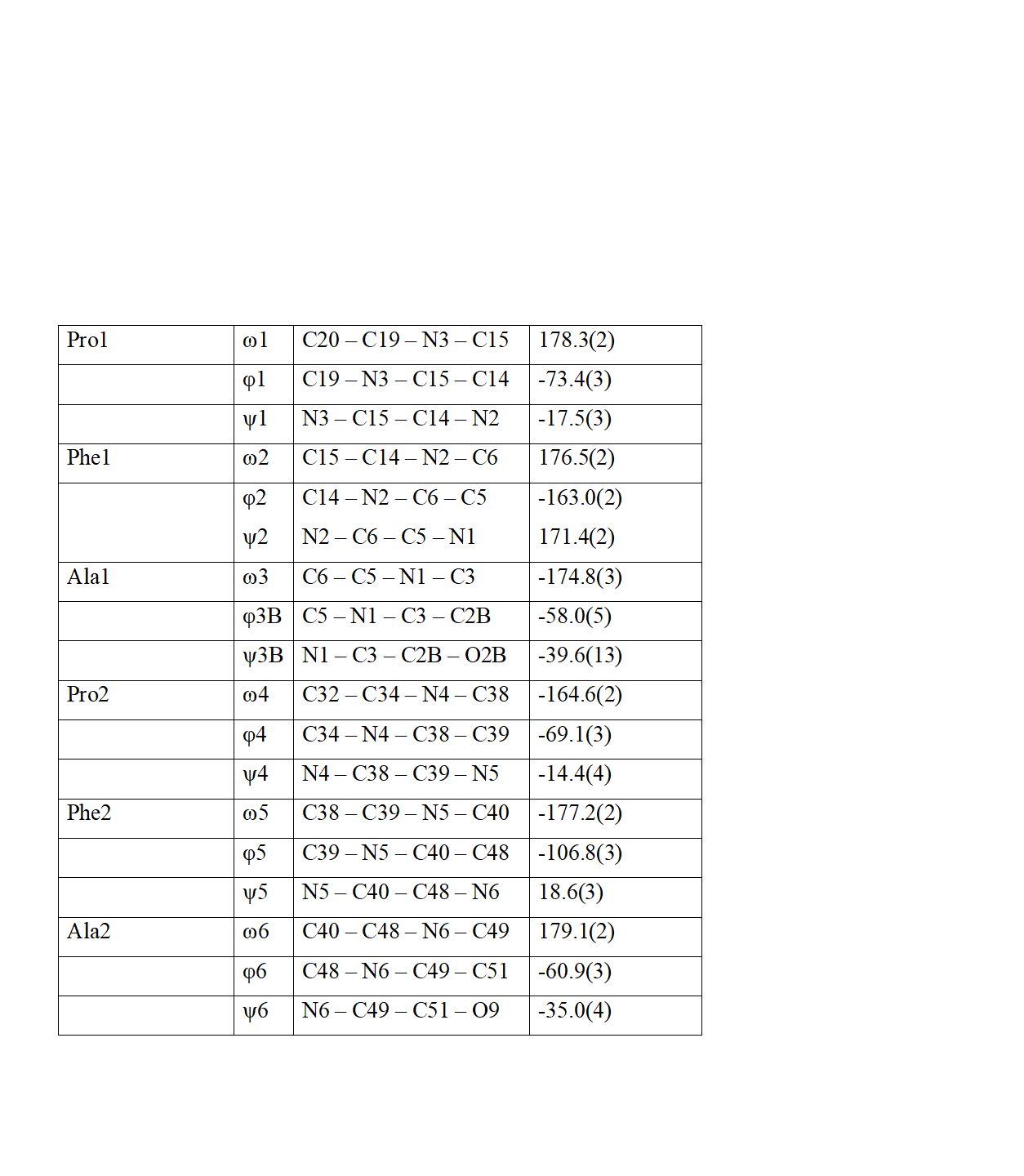

Supplement: Supplementary file 6 [file e-76-01675-sup7.tif]

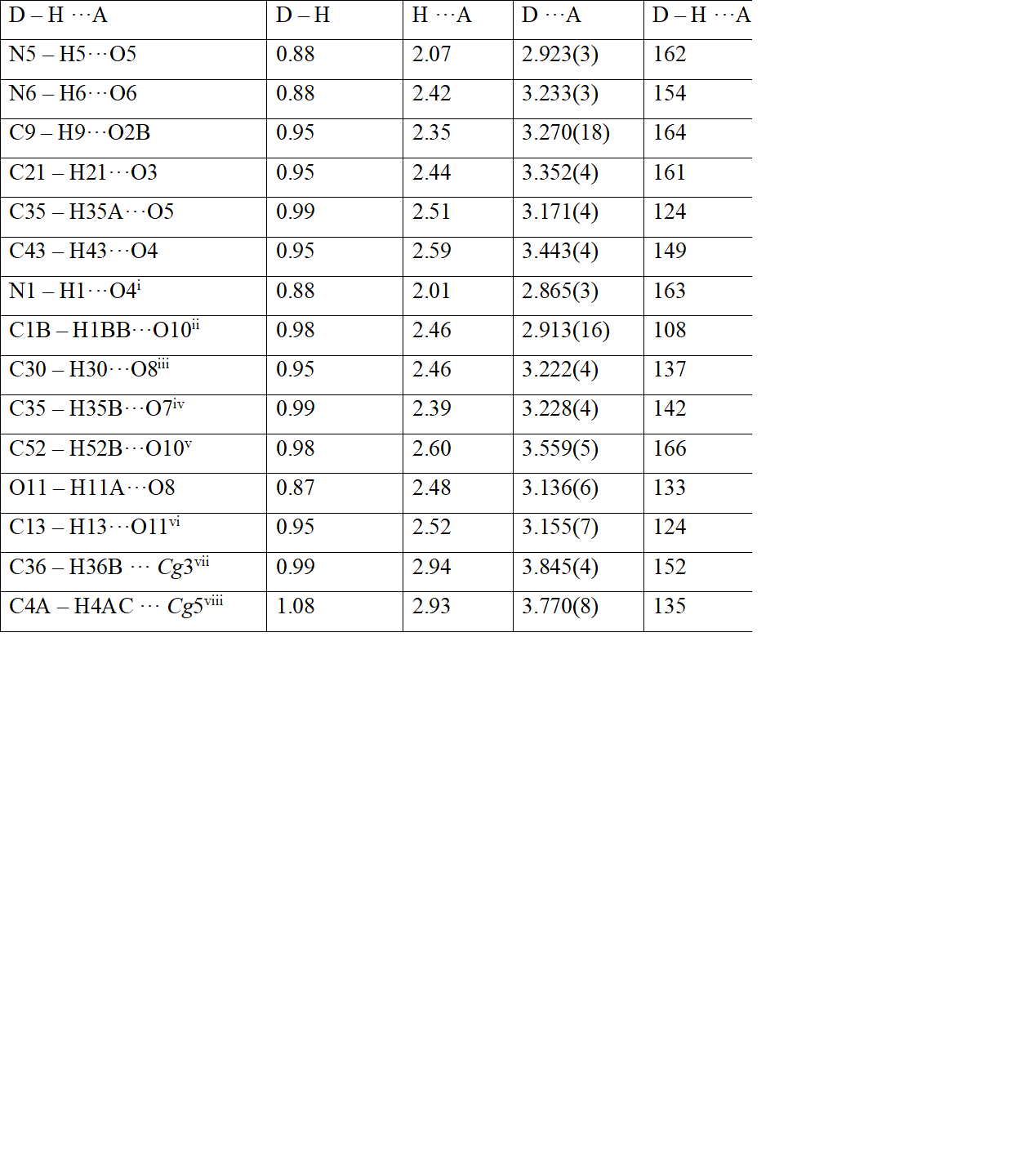

Supplement: Supplementary file 7 [file e-76-01675-sup8.tif]
